# Parvalbumin neurons mediate neurological phenotypes of anti-NMDAR encephalitis

**DOI:** 10.1093/brain/awae374

**Published:** 2025-03-12

**Authors:** Yi-Fan Feng, Zi-Ke Zeng, You Ni, Yue Hu, Ke-Xin Yang, Fang Cai, Qin-Ming Zhou, Ming Chen, Xiao-Na Zhu, Sheng Chen, Ji Hu

**Affiliations:** School of Life Science and Technology, ShanghaiTech University, Shanghai 201210, China; School of Life Science and Technology, ShanghaiTech University, Shanghai 201210, China; Department of Neurology and Institute of Neurology, Ruijin Hospital, Shanghai Jiao Tong University School of Medicine, Shanghai 200025, China; Department of Anesthesiology, Huashan Hospital, Fudan University, Shanghai 200040, China; School of Life Science and Technology, ShanghaiTech University, Shanghai 201210, China; School of Life Science and Technology, ShanghaiTech University, Shanghai 201210, China; Department of Neurology and Institute of Neurology, Ruijin Hospital, Shanghai Jiao Tong University School of Medicine, Shanghai 200025, China; MOE Frontier Center for Brain Science, Institutes of Brain Science, Fudan University, Shanghai 200032, China; School of Life Science and Technology, ShanghaiTech University, Shanghai 201210, China; Department of Neurology and Institute of Neurology, Ruijin Hospital, Shanghai Jiao Tong University School of Medicine, Shanghai 200025, China; Co-innovation Center of Neuroregeneration, Nantong University, Nantong 226019, China; School of Life Science and Technology, ShanghaiTech University, Shanghai 201210, China

**Keywords:** NMDAR, medial prefrontal cortex, parvalbumin neurons, cognitive impairments, gamma oscillations, rescue

## Abstract

Patients with anti-*N*-methyl-D-aspartate receptor (anti-NMDAR) encephalitis, often present with severe psychiatric symptoms, yet the neuropathological mechanisms underlying their cognitive deficits remain insufficiently understood. In this study, we constructed an animal model using anti-NMDAR IgG purified from the serum of patients with anti-NMDAR encephalitis, and we used IgG obtained from healthy individuals as a control. Daily administration of anti-NMDAR IgG into the medial prefrontal cortex (mPFC) of mice for 7 days resulted in cognitive impairments resembling clinical symptoms, which spontaneously resolved 30 days after discontinuing the injections.

Immunohistochemical staining and electrophysiological testing of parvalbumin neurons in the mPFC treated with anti-NMDAR IgG revealed significant cellular morphological damage, reduced excitability, synaptic dysfunction and a loss of NMDAR antagonist-induced gamma oscillations. Application of optogenetic and pharmacogenetic techniques to activate parvalbumin neurons in the mPFC successfully reversed the cognitive impairments observed in the anti-NMDAR-IgG-treated mice. Single-cell sequencing of anti-NMDAR-IgG-treated parvalbumin neurons identified differentially expressed genes and pathways related to synapses and neuronal development, offering potential targets for therapeutic intervention. Additionally, we showed that these alterations in parvalbumin neurons were not confined to the mPFC, as similar changes were detected in the hippocampus after anti-NMDAR IgG injections.

In summary, our findings elucidate distinct alterations in parvalbumin neurons during the pathogenesis of anti-NMDAR encephalitis, providing preclinical rationale for exploring approaches to modulate parvalbumin neuronal function to treat anti-NMDAR encephalitis.

## Teaser

Feng *et al.* have developed a mouse model to study anti-NMDAR encephalitis and its effects on brain function. They show that injecting anti-NMDAR antibodies into the medial prefrontal cortex of mice leads to cognitive impairments and dysfunction of parvalbumin-positive interneurons, mirroring changes observed in humans.

## Introduction

Anti-*N*-methyl-D-aspartate receptor (anti-NMDAR) encephalitis, also known as NMDAR-antibody encephalitis, is an autoimmune form of encephalitis in which the CSF or serum of patients contains anti-NMDAR IgG, resulting in a range of clinical symptoms including mental and cognitive impairment.^1,2^ Owing to its obscure aetiology, propensity for misdiagnosis, and poor prognosis, therapeutic options for anti-NMDAR encephalitis remain limited. Despite the increase in anti-NMDAR encephalitis research over the past decade and a half, there is a notable scarcity of in-depth studies exploring the neuropsychiatric mechanisms of this disease.^3,4^

Previous studies showed that cognitive decline is a common phenotype in anti-NMDAR encephalitis models.^5-7^ Planaguma *et al*.^5^ administered anti-NMDAR IgG into the lateral ventricles of mice over 2 weeks using osmotic pumps, which led to a reduction in NMDAR density in the hippocampus, accompanied by transient cognitive impairment and depressive symptoms, which resolved 3 weeks after the end of exposure. In fact, infiltration of autoimmune antibodies can affect various structures in the brain of patients with anti-NMDAR encephalitis. For example, functional MRI (fMRI) of patients showed abnormal temporal and hippocampal frontal signals.^3^ A few other studies have evaluated the ventricles and adjacent nuclei, such as the hippocampus,^7-9^ but the roles of other cognitive function-related brain areas, such as the medial prefrontal cortex (mPFC), in cognitive behavioural phenotypes related to anti-NMDAR encephalitis remain unexplored.

Studying the patterns of changes in excitatory neurons and inhibitory interneurons in anti-NMDAR encephalitis is important for elucidating its pathogenic mechanisms. Previous *in vitro* studies of cultured cortical neurons showed that anti-NMDAR IgG caused more damage to inhibitory interneurons than excitatory neurons.^10^ The mPFC harbours various types of interneurons, including parvalbumin (PV) neurons, somatostatin (SST) neurons and vasoactive intestinal peptide (VIP) neurons. Among them, PV neurons regulate gamma oscillations, which are highly associated with cognitive functions.^11-14^ Indeed, in psychiatric disorders, such as schizophrenia and autism, patients often exhibit impaired gamma oscillations.^15,16^ Additionally, antagonizing the NMDA receptors on PV neurons markedly affects their immunogenicity and results in functional impairments of the PV neurons, leading to cognitive deficits and other behavioural phenotypes.^17,18^ Notably, experimental evidence has shown that optogenetic activation of PV neurons, but not SST neurons, at 40 Hz improves social behavioural impairments. Furthermore, rescuing the activity of PV neurons using drugs can reverse the lipopolysaccharide (LPS)-induced attenuation of gamma oscillations and associated cognitive impairments.^13,19^ Based on these findings, we speculate that PV neurons and their NMDAR function may be closely associated with cognitive impairments in anti-NMDAR encephalitis.

In this study, we discovered the specific impairment of PV neurons by anti-NMDAR IgG in the mPFC and hippocampus. Furthermore, we demonstrated that activating mPFC PV neurons can reverse the cognitive impairments induced by anti-NMDAR IgG. Additionally, through single-cell sequencing, we identified numerous potential target genes and pathways to inform drug discovery priorities aimed at improving therapeutic options for patients with anti-NMDAR encephalitis.

## Materials and methods

### Animals

A total of 180 male C57BL/6J mice aged 6–8 weeks and 60 male PV-Cre mice aged 4–8 weeks were used in this study. C57BL/6J mice were purchased from Shanghai Jihui Experimental Animal Feeding Co., LTD., PV-ires-cre (Stock Number: 008069) mice were from Jackson Laboratory. The PV-ires-cre mice were identified according to the method provided by the Jackson Laboratory and primer information can be found on their website. The feeding environment of mice was maintained at 22–25 °C, the humidity was 40%–60%, the rhythm was 12-h/12-h day and night cycle, and the water and special food were of sufficient supply. Animal studies adhered to the ARRIVE Guidelines. This study was approved by the Ethics Committee of the ShanghaiTech University (Ethics No: 20200706002).

### Acquisition and purification of anti-NMDAR IgG

This experiment passed the ethical approval (Clinical project Ethics Approval Document No. 2021-375) of Ruijin Hospital and informed consent was received from the patients. Anti-NMDAR encephalitis patients from Ruijin Hospital showed abnormalities in their brain PET-MRI images (Supplementary Fig. 1A) and their CSF and serum antibody tests were positive for NMDAR only (Supplementary Fig. 1B). Patient details are provided in Supplementary Fig. 1C. The antibodies were pooled from the serum of four patients and used together after mixing. Peripheral blood was collected and purified by Protein G affinity chromatography at the Core Facility of Basic Medical Sciences of Shanghai Jiao Tong University School of Medicine.^20^ IgG concentration was determined by Nanodrop spectrophotometer.

### Stereotactic injection

As previously described,^21^ IgG antibodies derived from the patients or healthy individuals were injected into the brain area. The injection coordinates were as follows: medial prefrontal cortex: A/P: +1.67 mm, M/L: ±0.30 mm, D/V: −1.25 mm; hippocampus dCA1: A/P: −1.15 mm, M/L: ±1.50 mm, D/V: −1.70 mm. Antibodies (600 nl) were injected bilaterally into the mPFC or dorsal cornu ammonis subfields 1 (dCA1) daily, with the antibody concentration at 2 mg/ml, for seven consecutive days. After injection, the mice rested for at least 3 days prior to the behavioural and immunohistochemical staining experiments.

### Behavioural tests

All behavioural tests were conducted during the daily activity time of the experimental animals. The animals were adapted to the environment 2–3 h in advance to reduce the stress response.

#### Y-maze test

The mice were placed at the centre of the three-arm intersection of the Y-maze and were free to explore for 5 min. Cameras were used to record the movements of the mice. The proportion of the number of unrepeated shuttle explorations in the three arms relative to the total number of explorations was defined as ‘Alternation’.

#### Novel object recognition test

The method was described in a previous study.^22^ Cameras were used to record the movements of the mice. The time that the mice spent exploring the object was the time they spent smelling or touching it; the time they spent climbing it was not included. The ‘recognition index’ is defined as the ratio of the time spent exploring a novel object in relation to the total time spent exploring both new and familiar objects.

#### Three-chamber sociability test

The experiment consisted of three stages. In the first stage, empty cages were placed on both sides of the box and the mice were free to explore the box for 10 min. In the second stage, a strange, unknnown mouse was placed in a cage on one side of the box and the socialization time of the test mouse was automatically recorded by camera for 10 min. In the third stage, a cage mouse familiar to the test mouse was placed in the empty cage on the other side, and the socialization time of on both sides was recorded for 10 min. Social preference was defined as the time spent by the test mouse interacting with the unknown mouse during the second stage, divided by the total time spent with both mice and the empty cage. Social novelty is defined as the time spent by the test mouse interacting with the unknown mouse during the third stage, divided by the total time spent with both the unknown mouse and the familiar mouse.

#### Tail suspension test

The mice were suspended 20 cm from the ground with their heads down, for a total of 6 min. The first 2 min of suspension were discarded and the remaining 4 min of immobility were calculated. The ratio of immobile time/4 min was used as the index of depression.

#### Open field test

The mice were placed in a 38 × 38 × 38 cm chamber and allowed to move freely for a total of 10 min. Cameras were used to record the movements of the mice. MATLAB R2018b automatic video tracking system and data analysis were used to obtain the total motion distance of mice.

### Immunohistochemical staining

Prior to immunohistochemical staining, cardiac perfusion was performed using the method described in our previous study.^21^ The mice brains were then removed, soaked in 4% paraformaldehyde (PFA) at 4°C overnight, and transferred to 30% sucrose PBS solution for full dehydration for 48 h. Using the Leica^®^ CM3050S frozen microtome, continuous 40 μm slices were obtained along the coronal plane. The slices were incubated with 5% bovine serum albumin (BSA), 0.3% Triton-100 and 1× PBS solution at room temperature for 2 h to block non-specific protein binding. The primary antibody was added and incubated overnight, in a shaker, at 4°C. The secondary antibody was then selected according to the source of the primary antibody and the required fluorescence wavelength, and incubated at room temperature for 2 h away from light. For human IgG staining, the secondary antibody was 488 goat anti-human IgG (H+L) (1:1000, Invitrogen, A11013). For other immunohistochemical staining, the primary antibodies used were: goat anti-parvalbumin (1:1000, Swant, PVG-213), glial fibrillary acidic protein (GFAP) (1:1000, Proteintech, #16828-1-AP) and IBA-1 (1:1000, Wako, #019-19741). The secondary antibody used for these was 488 donkey anti-rabbit IgG (1:1000, Jackson ImmunoResearch).

Following immunohistochemical staining, the secondary antibody was washed with 1× PBS and the sample slides were sealed with 50% glycerol containing DAPI. An Olympus VS120 microscope and a Nikon rotary confocal microscope were used for observation.

### Confocal microscopy imaging and morphological analysis of neurons

All imaging experiments were performed at the Molecular Imaging Core Facility of School of Life Science and Technology of ShanghaiTech University.

The Nikon CSU SORA rotary confocal microscope (40× and 60× oil objectives) was used to realize *z*-stack and jigsaw mode to photograph the structure of the PV neurons. *Z*-axis steps were 0.2 μm. Each image slice was 1200 × 1200 pixels. Each channel used the same laser power and exposure time between experimental and control groups. Excitation/long-pass emission filters: Alexa Fluor^®^ 488 (excitation: 488 nm, emission: 493-584 nm filter), Alexa Fluor^®^ 405 (excitation: 405 nm, emission: 421 nm filter). According to previous studies,^1,23^ we used ImageJ to analyse morphological indexes such as neuron branch, dendrite length and Sholl analysis. The main body of the dendrite outward from the cell body is defined as the primary branch, and the first subsequent bifurcation is defined as the secondary branch. The total length of all dendrites extending from the cell body were calculated as the dendrite length. The Sholl analysis method has been described in previous studies.^24^

### *In vitro* electrophysiological experiment

The experiment included 18 C57B6/J mice and 24 PV-ires-cre mice. The PV-Cre mice were injected with AAV2/9-hSyn-DIO-mCherry (Brain VTA, Cat. No. PT-0115) or AAV2/9-hSyn -DIO-hChR2(H134R)-mCherry (Brain VTA, Cat. No. PT-0150) for 14 days to fluorescently label PV neurons *in vivo*. After 1 week of virus expression, the animals were divided into two groups: one group received 7 days of anti-NMDAR IgG injections, and the other group received 7 days of control IgG injections.

The mice were then euthanized by intraperitoneal injection of 625 mg/kg tribromoethanol and the brains were quickly removed and put into artificial CSF (in mM: 119 NaCl, 2.5 KCl, 2.5 CaCl_2_, 1.3 MgCl_2_, 26 NaHCO_3_, 1 NaH_2_PO_4_ with 95% O_2_/5% CO_2_) at 4°C, which was continuously oxygenated. After soaking for 1 min, the mice brains were transferred to an oscillating microtome equipped with ice tank precooled artificial CSF to obtain slices with a thickness of 300 μm. The slices were transferred to NMDG artificial CSF solution in a 31°C water bath and then transferred to artificial CSF in a 31°C water bath for 13 min and incubated for 1 h. Multiclamp 700b and pClamp were used to record the data. Data processing was performed using pClamp 10.0 and Mini Analysis 6.0.

In experiments measuring the paired pulse ratio (PPR) of inhibitory postsynaptic currents (IPSCs), two light pulses (2 ms duration), with an interval of 50 ms. were delivered to the mPFC every 30 s. The light intensity was adjusted to achieve IPSCs with an amplitude of 100–200 pA. The PPR was calculated as the ratio of the amplitude of the second IPSC over that of the first one.

To record NMDA currents, slices were bathed in the same artificial CSF solution used for storing the slices (without added MgCl_2_). Whole-cell NMDA currents were evoked by adding NMDA (50 uM) to the gravity-driven super fusion system, and rapid NMDA responses were obtained by first ‘cleaning’ the cell body, as described by Edwards *et al*.^25^

### *In vivo* electrophysiological experiment

Recording equipment was purchased from Qianao Xingke Nanjing Biotechnology Co., Ltd. The signal acquisition wire was 75 μm diameter stainless steel enamelled wire (Cat. No. 791000, A-M system). Electrode implantation was performed with a stereotactic device and the mice rested for 72 h after the operation. Data analysis was described in previous studies.^26^ Ephyslab software was used for recording. Sampling was carried out at 30 kHz sampling rate and data conversion was performed by Spike2 software (CED). Data analysis was performed by MATLAB 2018b. Power frequency noise (50 Hz) was filtered during data processing. The frequency segments in this study were: theta (4–8 Hz), alpha (8–12 Hz), beta (12–25 Hz), low-gamma (25–50 Hz) and high-gamma (50–80 Hz). In the MK-801 challenge experiment, we administered a 1-week injection of anti-NMDAR IgG or control IgG to the mPFC of mice, followed by an intraperitoneal injection of 0.3 mg/kg MK-801 half an hour after the local field potential (LFP) recording. Normalized power indicates power normalized by mean power during the baseline period, to highlight pre/post changes. During the novel object recognition (NOR) test, we selected a time window of 1 s before the mouse explored the novel object and 2 s after the exploration to analyse the energy of each frequency band. The normalized power change was defined as the mean power at every point divided by the mean of the baseline (1 s before the time window of the NOR event).

### Optogenetics experiment

A total of 14 PV-ires-cre mice were used for optogenetics. Two weeks before the optogenetics experiment, the experimental group was injected with AAV2/9-hSyn-DIO-hChR2(H134R)-mCherry virus in the mPFC (Brain VTA, Cat. No. PT-0150). One week later, they received anti-NMDAR IgG injections for an additional week. On the final day, optical fibres and LFP electrodes were placed in the target brain region. Coordinates and injection dose: A/P: +1.67 mm, M/L: ±0.30 mm, D/V: −1.25 mm, 200 nl; coordinates and angles of bilateral fibre: A/P: +1.67 mm, M/L: ±0.47 mm, D/V: −1.15 mm, 8°. The LFP recording electrode was wound on one side of the fibre to record the effect of optogenetics on the gamma oscillation led by PV neurons. Control mice were injected with AAV2/9-hSyn-DIO-mCherry (Brain VTA, Cat. No. PT-0115). Other operations were the same as the experimental group and received the same dose of light. We used an UNO single chip microcomputer to connect the computer MATLAB 2018b GUI operator and the laser transmitter, and the parameter settings are provided in previous studies.^27^ The light intensity was calculated to be 10 mW based on 473 nm blue light. The light stimulation mode was set to 40 Hz, 5 ms, 300 s, duty cycle to 20%. In the behavioural test, no light was given for the first 5 min and light was given for the last 5 min.

### Chemogenetics experiment

A total of 12 PV-ires-cre mice were used in pharmacological genetics experiments. Experimental mice were injected with AAV2/9-hSyn-DIO-hM3D(Gq)-mCherry (Brain VTA, Cat. No. PT-0019) virus. Control group mice were injected with AAV2/9-hSyn-DIO-mCherry (Brain VTA, Cat. No. PT-0115). One week after the virus injection, they received anti-NMDAR IgG injections for a week. Coordinates and dose of injection: A/P: +1.67 mm, M/L: ±0.30 mm, D/V: −1.25 mm, 200 nl. Thirty minutes before the start of behavioural experiment, the mice in each group received an intraperitoneal injection of 1 mg/kg CNO (Sigma-Aldrich, Cat. No. C0832).

### Single cell RNA sequencing, data processing and analysis

The single-nucleus RNA-seq experiment was performed at the laboratory of NovelBio Co., Ltd. The scRNA-Seq libraries were generated using the 10X Genomics Chromium Controller Instrument and Chromium Single Cell 3’ V3 Reagent Kits (10X Genomics). scRNA-seq data analysis was performed by NovelBio Co., Ltd., using the NovelBrain Cloud Analysis Platform. The Seurat package (version:4.1.1) was used for cell normalization and regression based on the expression table, according to the UMI counts of each sample and per cent of mitochondria rate to obtain the scaled data. Principal component analysis (PCA) was constructed based on the scaled data. The top 2000 high variable genes and top 10 principals were used for uniform manifold approximation and projection (UMAP) construction. Using the graph-based cluster method, we acquired the unsupervised cell cluster result based on the PCA top 10 principals and calculated the marker genes by FindAllMarkers. Significance was defined by the *P-*value and false discovery rate (FDR). To identify differentially expressed genes among samples, the function FindMarkers with Wilcox rank sum test algorithm was used under the following criteria: Log2FC > 0.25; *P-*value < 0.05; min.pct > 0.1.

For gene enrichment analysis, Fisher’s exact test was applied to calculate the *P-*value for each gene set. Raw *P-*values were adjusted for multiple hypothesis tests using the Benjamini and Hochberg method. Such enrichment analysis was applied to annotations including Gene Ontology (GO) and Kyoto Encyclopedia of Genes and Genomes (KEGG). To characterize the relative activation of a given gene set, we performed QuSAGE analysis.^28^

To enable a systematic analysis of cell-cell communication molecules, we applied cell communication analysis based on the CellPhoneDB,^29^ a public repository of ligands, receptors and their interactions. Membrane secreted and peripheral proteins of the cluster were annotated. Significant mean and cell communication significance (*P*-value < 0.05) was calculated based on the interaction and the normalized cell matrix achieved by Seurat normalization.

### Statistical analysis

All statistical analyses were processed by GraphPad Prism7 and MATLAB 2018b, with *P <0.05, **P < 0.01, ***P < 0.001 and ****P < 0.0001. Comparison between the two groups was performed using a *t*-test, multivariate ANOVA was performed using either one-way or two-way ANOVA, and all statistics were expressed using mean ± standard error of the mean (SEM).

## Results

### Anti-NMDAR IgG injected into the medial prefrontal cortex induces reversible cognitive impairment in mice

To confirm the long-term presence of anti-NMDAR IgG in the mPFC and its correlation with clinically relevant symptoms, we obtained and purified anti-NMDAR IgG from the serum of patients, and their PET data indicated significantly increased metabolism in the frontal, occipital and temporal lobes. Mini-Mental State Examination (MMSE) results demonstrated varying degrees of cognitive impairment in the patients (Supplementary Fig. 1). Accordingly, we designed a stereotactic injection model to precisely deliver the anti-NMDAR IgG to the mPFC for 7 days (Supplementary Fig. 2A). IgG isolated from healthy control subjects was injected into control mice. We then conducted cognition-related behavioural experiments to ascertain whether passive immunization of the mPFC of mice with patients’ purified anti-NMDAR IgG could recapitulate disease symptoms observed in humans. We found that anti-NMDAR IgG-treated mice displayed significant deficiencies during the Y-maze test and in NOR compared to control mice (Supplementary Fig. 2B and C), suggesting that anti-NMDAR IgG affected mPFC brain region function, leading to impairments in spatial cognition and working memory. However, no between-group differences were observed in the three-chamber social interaction test (Supplementary Fig. 2D), or in depression indicators (Supplementary Fig. 2E and F). To determine if antibody injections produced long-term effects in animals, we re-evaluated NOR and Y-maze performance 30 days after the 7-day treatment ended, and we found that anti-NMDAR-IgG-treated mice had recovered from cognitive function impairment (Supplementary Fig. 2G and H). Before and after antibody treatment, metabolic levels in both experimental and control groups remained unchanged (Supplementary Fig 2I–L), indicating that our modelling approach did not induce metabolic changes in the mice. Consistently, immunofluorescence staining indicated that, unlike control IgG, anti-NMDAR IgG persisted in the mPFC after 7 days of injection but disappeared naturally 1 month after treatment was stopped (Supplementary Fig. 3). Finally, we evaluated glia in the brain to determine whether these mice developed neuroinflammation. We observed specific activation of microglia 7 days after injection of anti-NMDAR IgG, but we did not observe intergroup differences in astrocytes or oligodendrocytes (Supplementary Fig. 4). Thus, we successfully established an animal model of anti-NMDAR encephalitis in the mPFC that is suitable for further investigations.

### Anti-NMDAR IgG causes morphological damage to parvalbumin neurons

**Figure 1:**
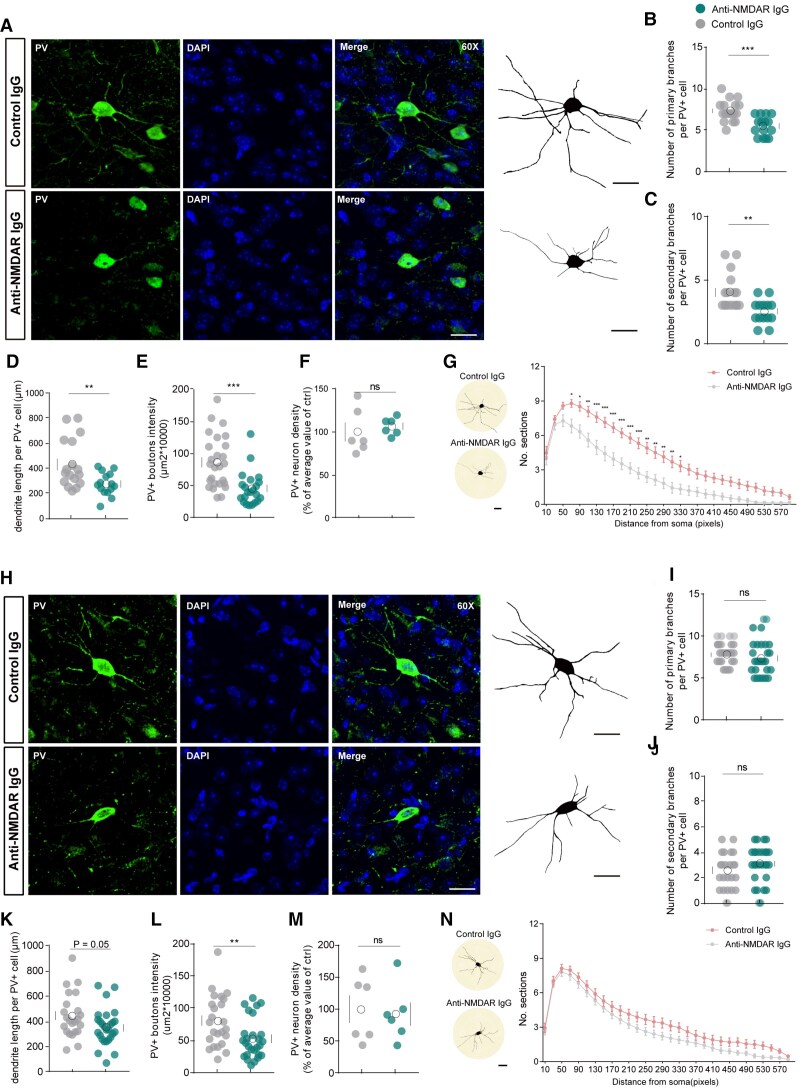
**The morphology of mPFC parvalbumin neurons was reversible after anti-NMDAR IgG injection**. (**A**–**G**) After 7 days of antibody injection, the morphology, scale bar = 20 μm (**A**), number of primary branches (**B**), number of secondary branches (**C**), dendrite length (**D**), PV+ boutons intensity (**E**), PV+ neuron density (**F**) and Sholl analysis (**G**) of medial prefrontal cortex (mPFC) PV neurons. Scale bar = 20 μm, *n* = 18–25 neurons of 6–7 mice. For PV+ neuron density (**F**), *n* = 6 mice. (**H**–**N**) Thirty days after antibody injection, the morphology, scale bar = 20 μm (**H**), number of primary branches (**I**), number of secondary branches (**J**), dendrite length (**K**), PV+ boutons intensity (**L**), PV+ neuron density (**M**) and Sholl analysis (**N**) of mPFC PV neurons. Scale bar = 20 μm, 1 pixel = 0.19 μm, *n* = 23–29 neurons of six mice. For PV+ neuron density (**M**), *n* = 6 mice. *P < 0.05, **P < 0.01, ***P < 0.001. ns = not significant; PV = parvalbumin.

Based on the observed cognitive impairments in our animal disease model, as well as previous research regarding the role of NMDAR on PV neurons in the mPFC in cognitive function,^30^ we investigated the neurophysiological mechanisms of anti-NMDAR IgG on PV neurons. After 7 days of anti-NMDAR IgG injection, immunofluorescence staining of PV neurons showed that the complexity of mPFC PV neurons decreased significantly (Fig. 1A). Specifically, compared to the control IgG injections, the mPFC PV neurons treated with anti-NMDAR IgG showed a marked reduction in the number of primary and secondary branches, reduced dendrite length of PV neurons, as well as reduced density of boutons indicative of PV neuron synapses (Fig. 1B–E). Although anti-NMDAR IgG had varying degrees of impact on indicators reflecting the complexity of neuronal structures, it did not affect the density of PV neurons (Fig. 1F). We then performed a Sholl analysis to statistically analyse the morphology of mPFC PV neurons after 7-day antibody injection, which showed a substantial decrease in dendritic complexity ∼20 μm from the cell body in anti-NMDAR-IgG-treated compared to control-IgG-treated PV neurons (Fig. 1G). These results suggest that 7 days of treatment with anti-NMDAR IgG caused damage to the morphology of PV neurons in the mPFC.

To determine whether the antibodies have a long-term impact at the neuronal function, we next conducted morphological analysis on PV neurons 30 days after the end of anti-NMDAR IgG injections. We found that there was a certain degree of incomplete recovery in PV neurons 30 days after injection. The complexity and branch count of PV neurons had been restored, while the density of PV boutons remained unchanged (Fig. 1H–M). The Sholl analysis indicated that, 30 days after antibody injection, the complexity of PV neurons treated with anti-NMDAR IgG was virtually indistinguishable from those treated with control IgG, suggesting partial recovery of PV neurons (Fig. 1N). These findings indicate that the structural damage to PV neurons caused by 7 days of anti-NMDAR IgG injection is partially reversible 30 days following the end of treatment.

### Anti-NMDAR IgG reduces synaptic function and gamma oscillation of parvalbumin neurons

**Figure 2:**
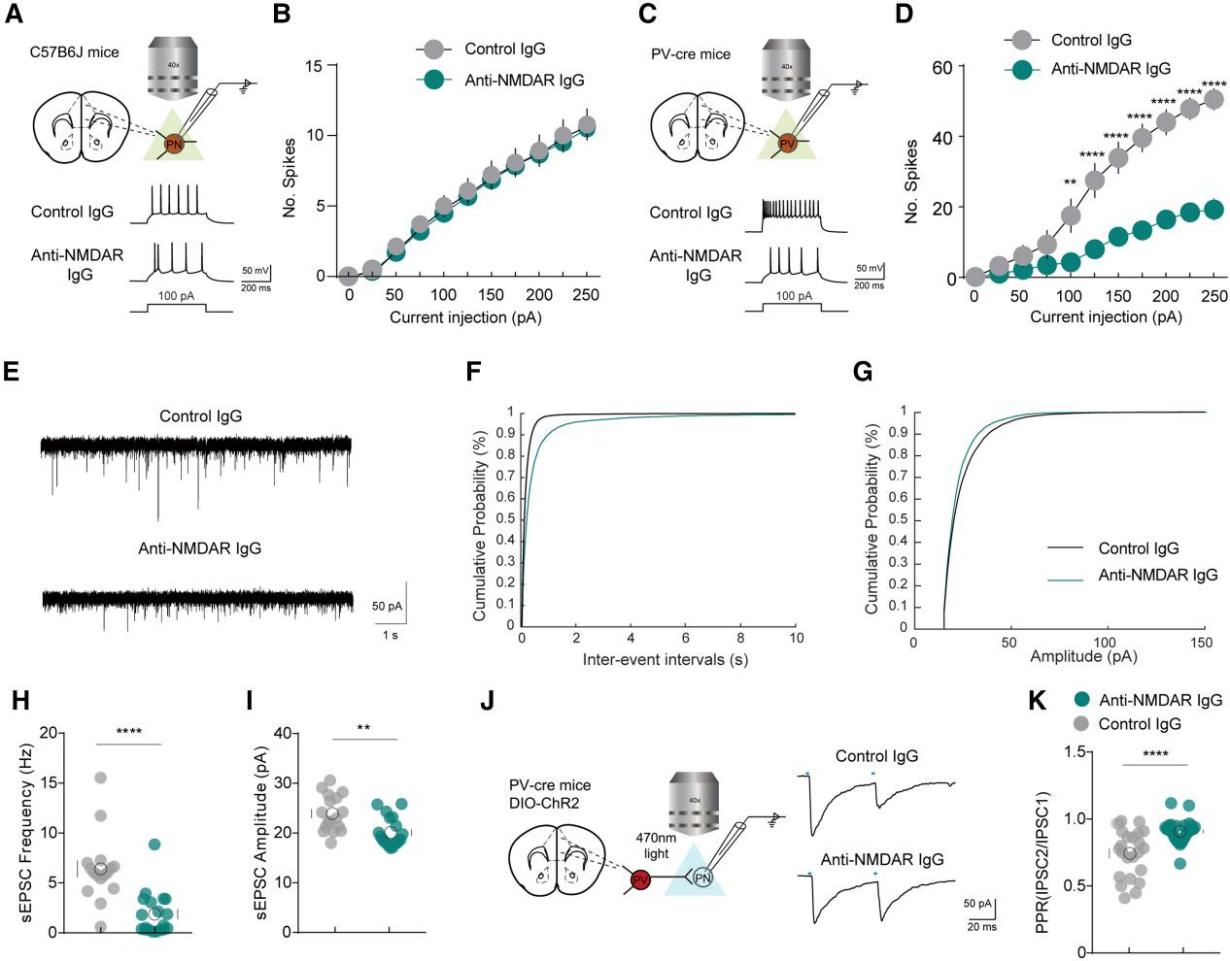
**Anti-NMDAR IgG specifically reduced the excitability of mPFC parvalbumin neurons and attenuated their inhibitory effect on pyramidal neurons**. (**A**) Schematic diagrams of patch-clamp recordings and evoked action potentials in pyramidal neurons of C57/B6J mice after 7 days of antibody injection. (**B**) Excitability of pyramidal neurons, *n* = 12 neurons from six mice. (**C**) Schematic diagrams of patch-clamp recordings and evoked action potentials in parvalbumin (PV) neurons of PV-Cre mice after 7 days of antibody injection. (**D**) Excitability of PV neurons, *n* = 12 neurons from six mice. **P < 0.01, ****P < 0.0001. (**E**) Representative traces of spontaneous excitatory postsynaptic currents (sEPSCs) of PV neurons, *n* = 16–19 neurons from six mice. (**F**) Cumulative probability plots of the inter-event interval of sEPSCs, analysed using Kruskal-Wallis one-way ANOVA. (**G**) Cumulative probability plots of the amplitude of sEPSCs, analysed using Kruskal-Wallis one-way ANOVA. (**H**) Average frequency of sEPSCs in mice injected with anti-NMDAR or control IgG. ****P < 0.0001. (**I**) Average amplitude of sEPSCs in mice injected with anti-NMDAR or control IgG. **P < 0.01. (**J**) Schematic diagram of paired pulse ratio (PPR) recordings in PV neurons after antibody injection. (**K**) Statistical analysis of PPR, *n* = 23–32 neurons from six mice. ****P < 0.0001.

To investigate whether anti-NMDAR IgG specifically affects neural activity of PV neurons, we initially examined the effects of antibodies on NMDA currents in mPFC neurons and found that NMDA currents in both PV and pyramidal neurons of mice injected with anti-NMDAR IgG were weaker than those in the control group (Supplementary Fig. 5A–D). Subsequently, we conducted patch-clamp recordings of neuronal excitability and, unexpectedly, we discovered that the excitability of mPFC PV neurons in mice receiving anti-NMDAR IgG injections was lower than that of the control group, whereas no difference was observed in pyramidal neurons (Fig. 2A–D). This suggests that the excitability of PV neurons is relatively more suppressed by anti-NMDAR IgG compared to that of pyramidal neurons. We also investigated the excitatory synaptic transmission in PV neurons and found that both the amplitude and frequency of spontaneous excitatory postsynaptic currents (sEPSCs) in PV neurons were reduced (Fig. 2E–I). Additionally, we measured the spontaneous IPSCs (sIPSCs) of pyramidal neurons. The data showed a decrease in IPSC frequency with no change in amplitude in anti-NMDAR-IgG-treated compared to control-IgG-treated neurons (Supplementary Fig 5E–I), indicating that presynaptic inhibitory inputs to pyramidal neurons are significantly reduced under the influence of anti-NMDAR IgG. To verify the inhibitory effect of PV neurons—which are a major source of inhibition for pyramidal neurons—on local pyramidal neurons, we specifically examined the synaptic transmission capabilities of mPFC PV neurons to local pyramidal neurons, which showed that the inhibitory effect of mPFC PV neurons from mice injected with anti-NMDAR IgG on local pyramidal neurons was diminished compared to controls (Fig. 2J and K). This confirmed our hypothesis of weakened output capability in PV neurons due to anti-NMDAR IgG treatment and also confirmed a marked reduction in PV neurons’ downstream inhibitory ability.

**Figure 3:**
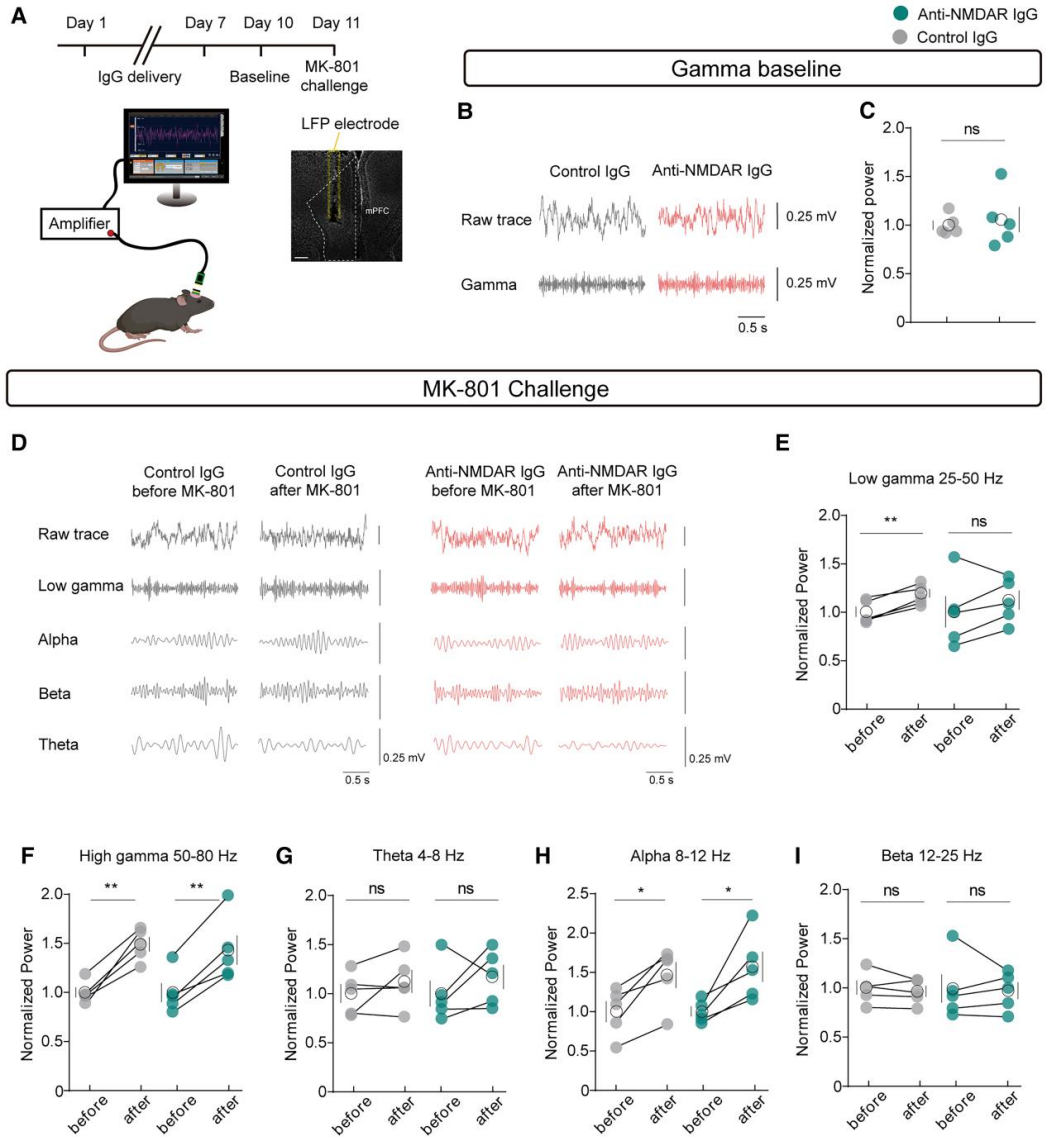
**Anti-NMDAR IgG abolished the NMDAR antagonist-induced elevation of low gamma oscillations**. (**A**) Schematic diagram showing the timeline for MK-801 challenge and local field potential (LFP) electrode position. Scale bar = 200 μm. (**B** and **C**) Gamma baseline without MK-801 injection, *n* = 5. (**D**–**I**) Comparison of oscillations in various frequency bands before and after intraperitoneal injection of MK-801. (**D**) Example LFP data of mice. (**E**–**I**) Statistical analysis of normalized LFP power in the low gamma, high gamma, theta, alpha and beta bands. *n* = 5, *P < 0.05, **P < 0.01.

Given that gamma oscillations, which are highly related to cognitive abilities, are regulated by PV neurons, we then performed *in vivo* local field potential (LFP) recordings (Fig. 3A). Baseline gamma, theta, alpha and beta oscillations remained unchanged in both treatment groups (Fig. 3B and C and Supplementary Fig. 6). However, in oscillations induced by the NMDAR antagonist MK-801, mice receiving anti-NMDAR IgG did not show a significant increase, specifically in the low gamma band (25–50 Hz), whereas their performance remained consistent with the control group across other frequency bands, suggesting an antibody-induced impairment of NMDAR function in mPFC PV neurons that selectively affects gamma oscillations (Fig. 3D–I).

### Activation of medial prefrontal cortex parvalbumin neurons reverses cognitive dysfunction by anti-NMDAR IgG

**Figure 4:**
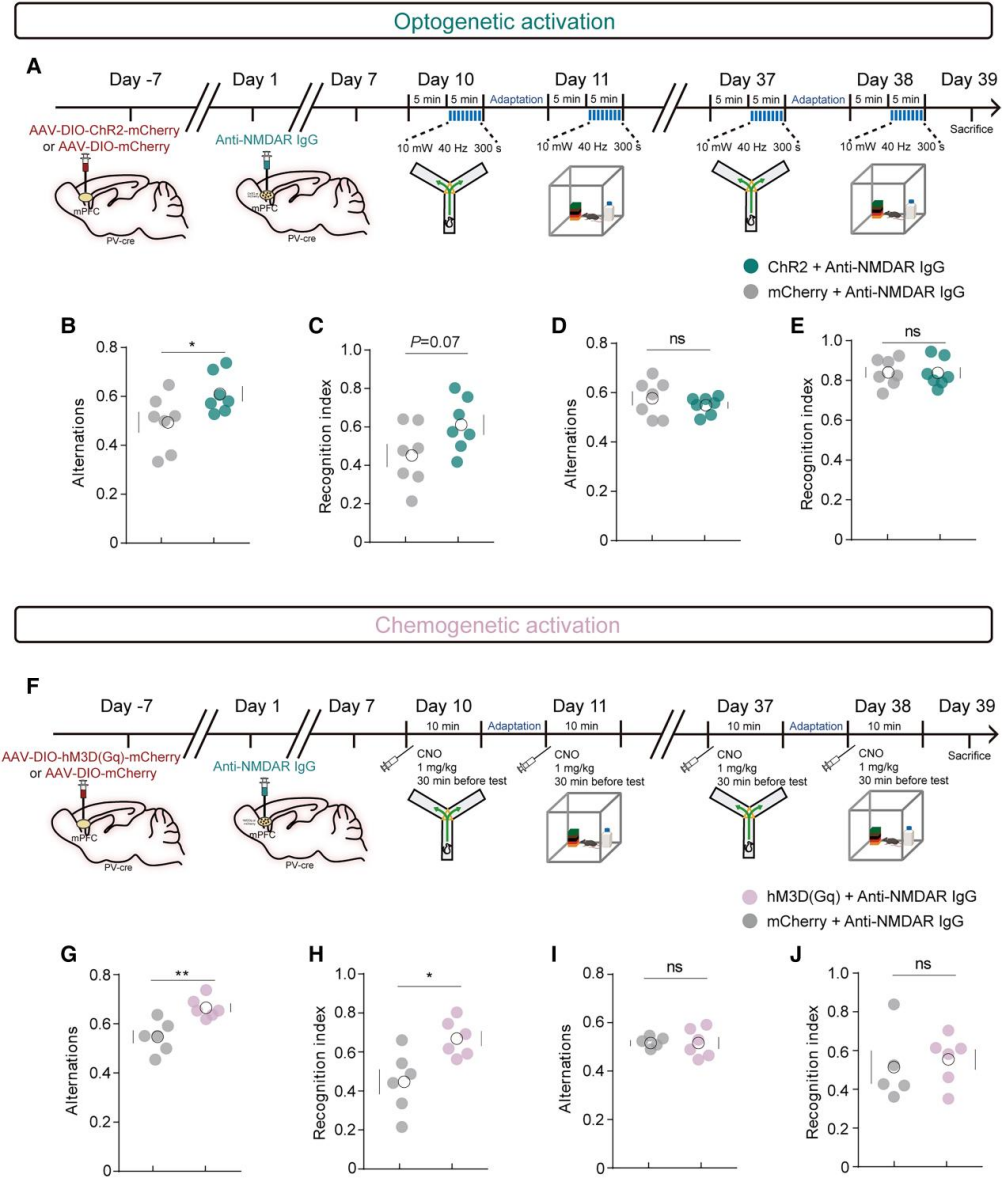
**Activation of mPFC parvalbumin neurons reversed cognitive impairment in mice injected with anti-NMDAR IgG**. (**A**) Schematic diagram showing the timeline for optogenetic behavioural experiments. (**B** and **C**) Optogenetic activation of the medial prefrontal cortex (mPFC) PV neurons on Y-maze and novel object recognition (NOR) tests in mice after 7 days of antibody injection. *n* = 7, *P < 0.05. (**D** and **E**) Optogenetic activation of the mPFC PV on Y-maze and NOR tests in mice 30 days after antibody injection. *n* = 7. (**F**) Schematic diagram showing the timeline for chemogenetic behavioural experiments. (**G** and **H**) Chemogenetic activation of the mPFC PV neurons on Y-maze and NOR tests in mice after 7 days of antibody injection. *n* = 6, *P < 0.05, **P < 0.01. (**I** and **J**) Chemogenetic activation of the mPFC PV neurons on Y-maze and NOR tests in mice 30 days after antibody injection. *n* = 5–6.

To explore the potential of mPFC PV neurons as therapeutic targets in the anti-NMDAR encephalitis animal model, we injected photogenetic [AAV2/9-hSyn-DIO-hChR2(H134R)-mCherry or AAV2/9-hSyn-DIO-mCherry] or chemogenetic [AAV2/9-hSyn-DIO-hM3D(Gq)-mCherry or AAV2/9-hSyn-DIO-mCherry] viruses into the mPFC of PV-Cre mice and continued antibody injections to observe the effects of selective activation of PV neurons on cognitive behaviour (Fig. 4A and F). Fibres and LFP recording electrodes were simultaneously implanted in the mPFC of mice (Supplementary Fig. 7A). During blue light stimulation, an immediate increase in gamma band energy was recorded in the ChR2 group, with no increase observed in the control group (Supplementary Fig. 7B). After 7 days of antibody injection, ChR2-expressing mice performed better cognitively than control mice in the Y-maze and NOR tests (Fig. 4B and C). Thirty days after antibody injection, light activation had no significant effect on cognitive behaviour in either group (Fig. 4D and E). Consistent results were observed when using the chemogenetic viruses. Specifically, after 7 days of antibody injection, mice administered 1 mg/kg Clozapine-*N*-oxide (CNO) intraperitoneally 30 min before the experiment and expressing hM3D(Gq), outperformed controls in the Y-maze and NOR tests (Fig. 4G and H and Supplementary Fig. 7C), and 30 days after antibody injection, pharmacological activation had no significant effect on cognitive behaviour in either group (Fig. 4I and J). These results suggest that activation of mPFC PV neurons through optogenetic or chemogenetic means can effectively ameliorate the cognitive impairment caused by anti-NMDAR IgG.

### Single-cell sequencing reveals the impact of anti-NMDAR IgG on gene and signalling pathways of parvalbumin neurons

**Figure 5:**
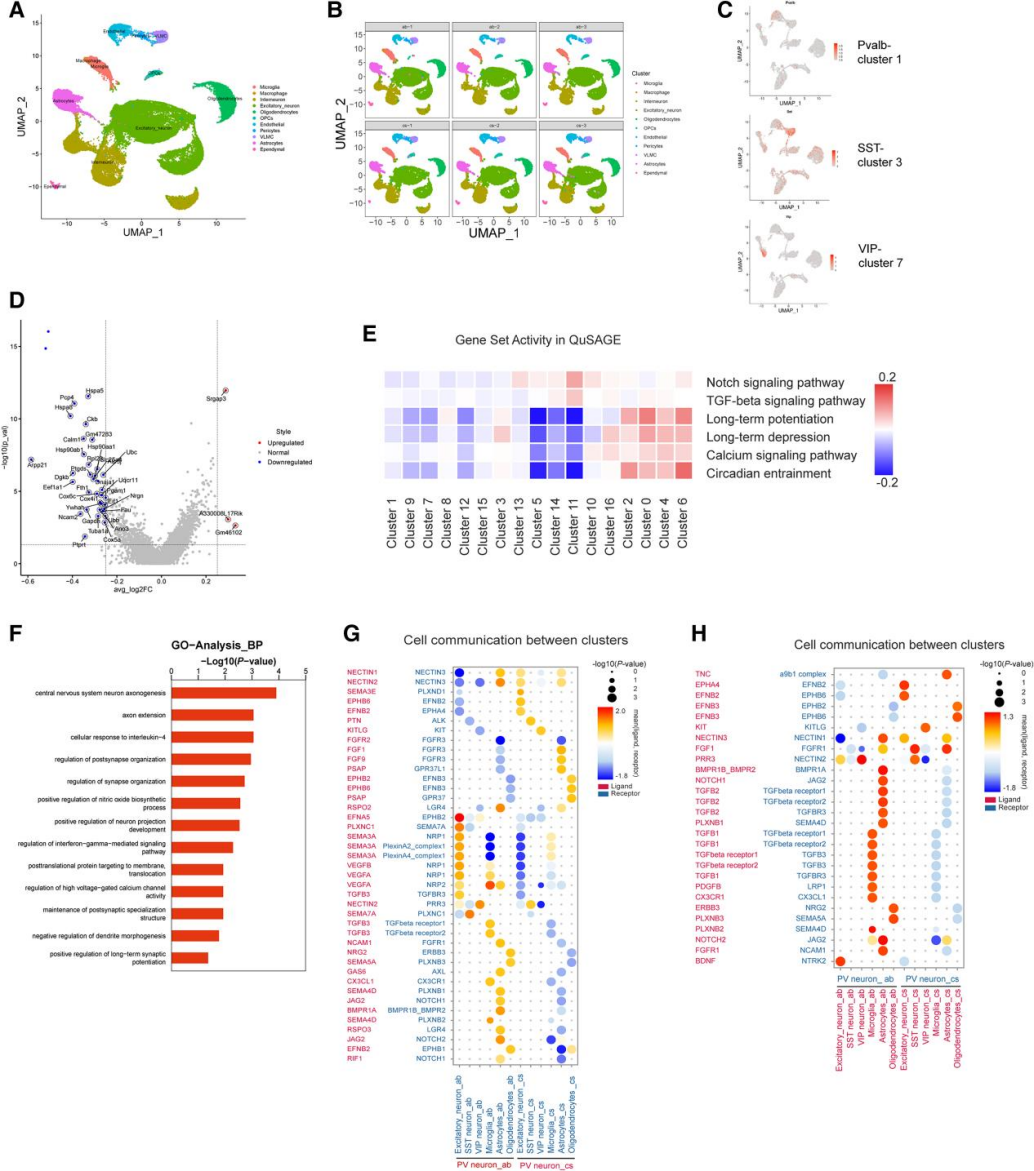
**Single-cell sequencing after injection of control IgG/anti-NMDAR IgG into the medial prefrontal cortex**. (**A** and **B**) A uniform manifold approximation and projection (UMAP) displaying cells from the medial prefrontal cortex (mPFC), categorized into 11 cell types using recognized markers and highly enriched genes. (**C**) Identification of major neurons in the mPFC. Pvalb = parvalbumin (PV) neurons; SST = somatostatin neurons; VIP = vasoactive intestinal peptide neurons. (**D**) Volcano map of differentially expressed genes between the anti-NMDAR IgG group and control IgG group. (**E**) Differentially expressed signalling pathways in each cluster. (**F**) Top signalling pathway enriched in Gene Ontology (GO) analysis. (**G** and **H**) Cell communication between clusters when PV neurons act as ligands (**G**) and receptors (**H**), *n* = 3.

To delve deeper into the potential molecular mechanisms affected by anti-NMDAR IgG within PV neurons, we investigated the genetic alterations in PV neurons within the mPFC at the single-cell level. We injected either anti-NMDAR IgG or control IgG into the mPFC of mice and divided them into two groups for subsequent sequencing. Cluster analysis identified various neuronal clusters in the mPFC using genes such as parvalbumin (*Pvalb*), somatostatin (*Sst*) and vasoactive intestinal peptide (*Vip*) as targets (Fig. 5A–C). We observed numerous differentially expressed genes (DEGs) in the mPFC PV neurons treated with anti-NMDAR IgG compared to those treated with control IgG (Fig. 5D); many were associated with synaptic and neuronal development, such as *Calm1*, *Ncam2*, *Nrgn* and *Eef1a1*, among others. Notably, gene sets downregulated in the anti-NMDAR IgG group were predominantly associated with synaptic and developmental pathways (Fig. 5E). Subsequent GO pathway analysis indicated downregulation in pathways related to NMDAR, postsynaptic function and axonal development within the gene clusters of anti-NMDAR IgG-treated PVs, the top four of which were CNS neuron axonogenesis, axon extension, cellular response to interleukin-4, and regulation of postsynapse and synapse organization (Fig. 5F). Last, cell phone analysis in which PV neurons acted as receptors showed enhanced communication between PV neurons and glial cells caused by anti-NMDAR IgG treatment, indicating inflammation, while expression of genes involved in neural repair were upregulated between PV and pyramidal neurons, although synaptic-related genes, such as *Nectin* and *Ephb2*, were downregulated in the anti-NMDAR-IgG-injected group compared to the control group (Fig. 5G and H). These findings highlight changes in the strength of neuronal communication via PV neurons under the influence of anti-NMDAR IgG.

### Anti-NMDAR IgG injected into the dCA1 causes cognitive dysfunction

Given the pivotal role of PV interneurons under the influence of anti-NMDAR IgG in cognitive deficits observed in our anti-NMDAR encephalitis mouse model, we investigated whether this effect was specific to this brain region. We continuously injected anti-NMDAR IgG into the hippocampal dCA1 region and found that anti-NMDAR IgG could induce reversible cognitive impairments and neuroinflammation (Supplementary Figs 8 and 9), consistent with our findings of mice treated with anti-NMDAR IgG in the mPFC. Further, *in vivo* electrophysiological recordings showed that, during the NOR task, mice injected with anti-NMDAR IgG into dCA1 exhibited significantly lower gamma oscillations while sniffing new objects compared to the control group, with other frequency bands unaffected. Thirty days after the antibody injections ceased, this phenomenon spontaneously reversed in anti-NMDAR-IgG-treated mice, suggesting that the changes in gamma oscillations were reversible (Supplementary Fig. 10). We also identified morphological impairments to PV neurons in the hippocampal dCA1 region caused by anti-NMDAR IgG, demonstrating that the effects of anti-NMDAR IgG on PV neurons are not limited to the mPFC (Supplementary Fig. 11).

## Discussion

In this study, we established an animal model of anti-NMDAR encephalitis in the mPFC. Using this model, we observed unique impairments of PV neurons caused by anti-NMDAR IgG, including disruptions in neuronal morphology, synaptic plasticity and gamma oscillations. Furthermore, we successfully reversed the cognitive impairments induced by these antibodies through the application of optogenetic and pharmacogenetic techniques. Ultimately, our investigation provides crucial insights into the neuromolecular mechanisms underlying cognitive impairments in anti-NMDAR encephalitis at the single-cell level.

The association between NMDAR and cognitive function has been extensively studied, particularly in cortical brain regions. Animal studies have shown that specific damage or inhibition of cortical NMDARs can lead to severe cognitive impairments,^30-32^ and cognitive impairments and frontal lobe dysfunction have also been observed in some patients with anti-NMDAR encephalitis.^3^ However, the pathogenic mechanism of anti-NMDAR encephalitis antibodies in the prefrontal cortex remains unclear. The cognitive impairments, reduced NMDA-induced currents, and inhibitory effects on PV neurons observed in our model are similar to the effects of direct NMDAR antagonist injection.^33,34^ Previous studies confirmed that anti-NMDAR IgG can induce reversible NMDAR internalization and exhibit spontaneous self-recovery over time,^5,35^ which aligns with two other key experimental findings of this study: 30 days after anti-NMDAR IgG injection, mice fully recovered from the cognitive impairments exhibited behaviourally and PV neuron damage also largely recovered; at the same time, optogenetic and chemogenetic manipulations were unable to improve behaviour in both groups of mice previously injected with anti-NMDAR IgG 30 days after treatment. Our study confirms that NMDARs in the mPFC indeed play a role in causing the cognitive impairment phenotype of anti-NMDAR encephalitis, which is crucial knowledge to inform clinical approaches targeting specific brain regions in autoimmune brain diseases.

PV neurons are a small but critical group of interneurons in the mPFC, comprising only ∼2% of the total neuronal population.^36^ They participate in forming cortical neural oscillations and maintaining the excitatory-inhibitory balance in the frontal lobe, and they are involved in important functions, such as cognition and addiction.^14,37,38^ When NMDAR antagonists act on all neurons in the mPFC, they preferentially inhibit interneurons, such as PV neurons, causing cortical excitation and changes in gamma oscillations governed by PV neurons.^39,40^ Our patch-clamp results suggest that mPFC PV neurons exhibit different firing patterns from pyramidal neurons under the influence of anti-NMDAR IgG. In the present study, we focused on mPFC PV neuron morphology and conducted immunohistochemical staining, and our results reflect a decline in the ability of PV neurons to receive upstream information and transmit downstream information, corroborating the electrophysiological data that showed weakened inhibitory output of PV neurons to pyramidal neurons.

Recent studies indicated that intraperitoneal injection of the NMDAR antagonist MK-801 can activate cortical gamma oscillations. However, in mice with specific knockout of NMDAR1 in PV neurons, intraperitoneal injection of MK-801 fails to activate gamma oscillations at 30–50 Hz.^30^ This observation aligns with the results obtained from mice injected with MK-801 after 7 days of anti-NMDAR IgG injection, providing compelling evidence that the NMDAR receptors on PV neurons are inhibited. Another study showed that, under the influence of anti-NMDAR IgG, cortical interneurons become less sensitive to NMDAR antagonists,^10^ likely due to reduced sensitivity of PV neurons to NMDAR antagonists, which also aligns with our observations. Nevertheless, no changes in baseline gamma oscillations were observed upon anti-NMDAR IgG treatment, which we hypothesize may be because inhibition of NMDAR on PV neurons by anti-NMDAR IgG may not reach the same intensity as genetic knockout. It is worth mentioning that we did not observe effects of anti-NMDAR IgG on social behaviours in the mPFC, possibly because the antibodies did not affect the somatic morphology of mPFC PV neurons or the function of pyramidal neurons, nor did they cause changes in PV neuron numbers. Experiments related to PV neuron-associated social deficits typically presuppose somatic damage to PV neurons,^27,41^ and studies have shown that selective inhibition of mPFC PV neurons does not cause social deficits.^11^ Therefore, due to the different mechanisms of action of antibodies compared to other small molecules or receptor modification mechanisms, we think that social behaviour is likely not severely affected by anti-NMDAR IgG in the mPFC.

As PV neurons play a critical role in cognitive function, we hypothesized that activating PV neurons might rescue the cognitive deficits caused by anti-NMDAR IgG. Previous research demonstrated that restoring the excitatory-inhibitory balance in the mPFC can effectively improve cognitive and social abilities in mice.^42,43^ Additionally, selective activation of mPFC PV neurons has been shown to ameliorate cognitive impairments caused by various diseases.^44,45^ Besides, optogenetic stimulation of PV interneurons at gamma frequency (40 Hz) rather than other frequencies, has a therapeutic effect on cognitive impairments in Alzheimer’s disease.^46^ We used optogenetic and chemogenetic techniques to activate mPFC PV neurons, effectively rescuing the cognitive deficits induced by anti-NMDAR IgG. The fact that PV neuronal activation was effective only after anti-NMDAR IgG exposure and did not improve behaviour 30 days after treatment ended suggests that the behavioural impairments caused by the anti-NMDAR IgG had fully recovered by that time.

In our single-cell sequencing analysis, we observed downregulation of genes associated with synaptic plasticity and neuronal development in mPFC PV neurons treated with anti-NMDAR IgG. Notably, expression of *Calm1* has been reported to be related to axon length^47^; *Eef1a1* to synaptic structure maintenance and axon repair^48,49^; and *Pgam1* to preventing neuronal damage induced by oxidative stress or ischaemia.^50,51^ These DEGs are strong candidates to prioritize for future studies aiming to enhance their expression to improve neuronal function. QuSAGE analysis revealed downregulation of signalling pathways in anti-NMDAR-IgG-treated PV neurons, including NOTCH, TGF-β, long-term potentiation (LTP), long-term depression (LTD), calcium and circadian entrainment. The NOTCH and TGF-β signalling pathways play crucial roles in neuronal development and synaptic plasticity.^52-54^ Downregulation of LTP and LTD suggests impaired synaptic plasticity, affecting adaptability and learning of the neural network.^55^ Moreover, dysregulation of calcium signalling may impact neural communication and synaptic plasticity, contributing to cognitive impairments.^56^ Downregulation of the circadian entrainment pathway is also noteworthy, as disruptions in circadian rhythms affect neural activity temporal organization and cognitive function.^57^ GO pathway analysis confirmed impairments in PV neuron morphology and synaptic function, and cell phone analysis revealed enhanced PV neuron-glial cell interactions and weakened PV neuron-excitatory neuron interactions. These unique changes offer potential for identification of novel drug targets to modulate these pathways, considering the intricate connections between PV neurons and their surrounding neurons.

We demonstrated a unique inhibition of mPFC-PV by anti-NMDAR IgG, but whether this phenomenon exhibits brain region specificity remained unclear. Therefore, we adopted a similar strategy to evaluate the effect of anti-NMDAR IgG on dCA1. We determined that anti-NMDAR IgG administered into the hippocampus induced cognitive behavioural impairments, accompanied by alterations in gamma oscillations during behaviour tests. Additionally, we observed changes in the morphology of PV neurons. These findings suggest that the impact of anti-NMDAR IgG on PV neurons may be concomitant with antibody distribution, implying that targeted drugs may be most effective if aimed at NMDAR of PV neurons across the entire brain.

Our findings underscore the significant impact of anti-NMDAR IgG not only on PV neurons in the prefrontal cortex but also in the hippocampus, suggesting a widespread influence of these antibodies in the progression of anti-NMDAR encephalitis. A critical role of PV neurons was discovered in the mechanisms through which anti-NMDAR IgG elicits cognitive phenotypes associated with anti-NMDAR encephalitis. We also propose that manipulating PV neurons could be a novel therapeutic strategy to treat anti-NMDAR encephalitis and advocate for further research into the role of PV neurons in this condition.

## Supplementary Material

awae374_Supplementary_Data

## Data Availability

The data and code that support the findings of this study are available from the corresponding author, upon reasonable request.
