# Supplementary material for: Parvalbumin neurons mediate neurological phenotypes of anti-NMDAR encephalitis
Source: Brain. 2025 Mar 12;148(5):1652–64. doi: 10.1093/brain/awae374 (PMC12073974; doi:10.1093/brain/awae374)
Supplement: awae374_Supplementary_Data [file awae374_supplementary_data.pdf]

Supplementary material

Table of Contents

Figure S1 Clinical information of anti-NMDAR encephalitis patients

Figure S2 Injections of control/anti-NMDAR IgG in the medial prefrontal cortex led to cognitive impairment in mice, without affecting their metabolic status

Figure S3 Binding of the anti-NMDAR IgG to the brain region is reversible

Figure S4 The inflammatory impact induced by anti-NMDAR IgG injection in the mPFC

Figure S5 Anti-NMDAR IgG attenuates inhibitory inputs to mPFC pyramidal neurons and equivalently reduces NMDA currents in both pyramidal and PV neurons

Figure S6 Injection of anti-NMDAR IgG did not result in statistically significant differences in theta, alpha, or beta frequency bands

Figure S7 Validation of the expression of optogenetic and pharmacogenetic viruses

Figure S8 Behavioral test results after injection of anti-NMDAR IgG in the dCA1 region

Figure S9 Injection of anti-NMDAR IgG for seven days in the CA1 region led to neuroinflammation

Figure S10 The injection of anti-NMDAR IgG in the hippocampus dCA1 caused cognitive impairment and decreased gamma oscillations

Figure S11 The injection of anti-NMDAR IgG in the hippocampus dCA1 caused changes in PV neurons

A

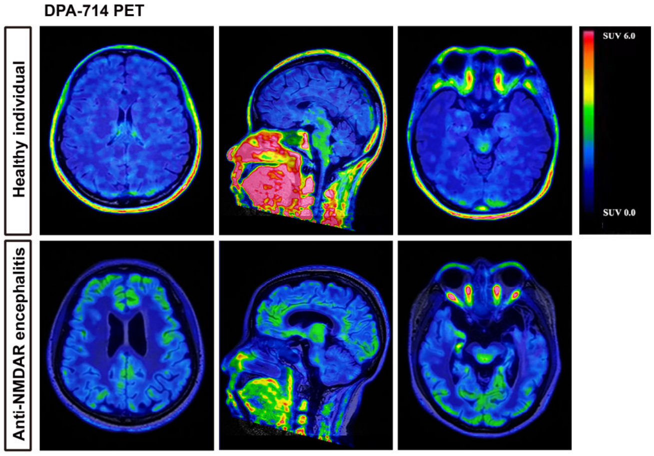

B

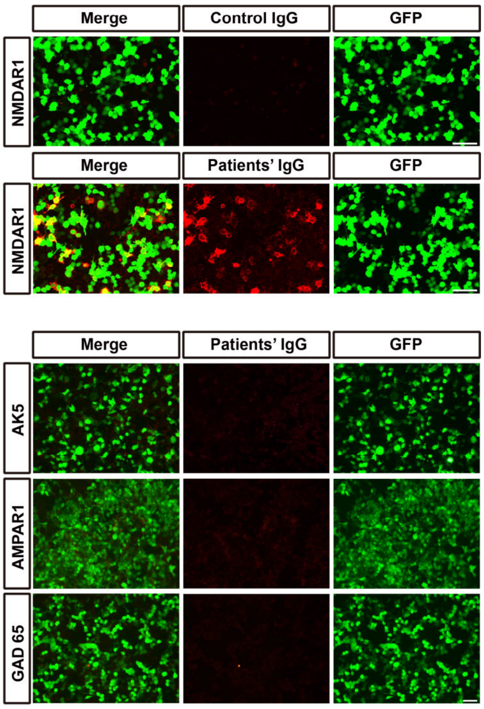

C

Table				
Patients	Gender	Age	Titer	MMSE
1	male	36	1:10	16
2	female	25	1:100	23
3	female	31	1:10	15
4	male	25	1:10	23

Figure S1: Clinical information of anti-NMDAR encephalitis patients. (**A**) The PET-MR image of a patient and healthy control. The standardized uptake value ratios (SUVR) of the left frontal lobe, right frontal lobe, left temporal lobe, right temporal lobe, left occipital lobe and right occipital lobe are 1.56, 1.59, 1.20, 1.52, 1.78, and 1.77, respectively. (**B**) Representative images of cell-based assays show that there are no NMDAR antibodies in the serum of healthy controls, whereas only anti-NMDAR antibodies are present in the patient's serum. Scale bar = 100 μm. (**C**) Patient information table, including gender, age, antibody titer, and cognitive level. MMSE = Mini-mental State Examination.

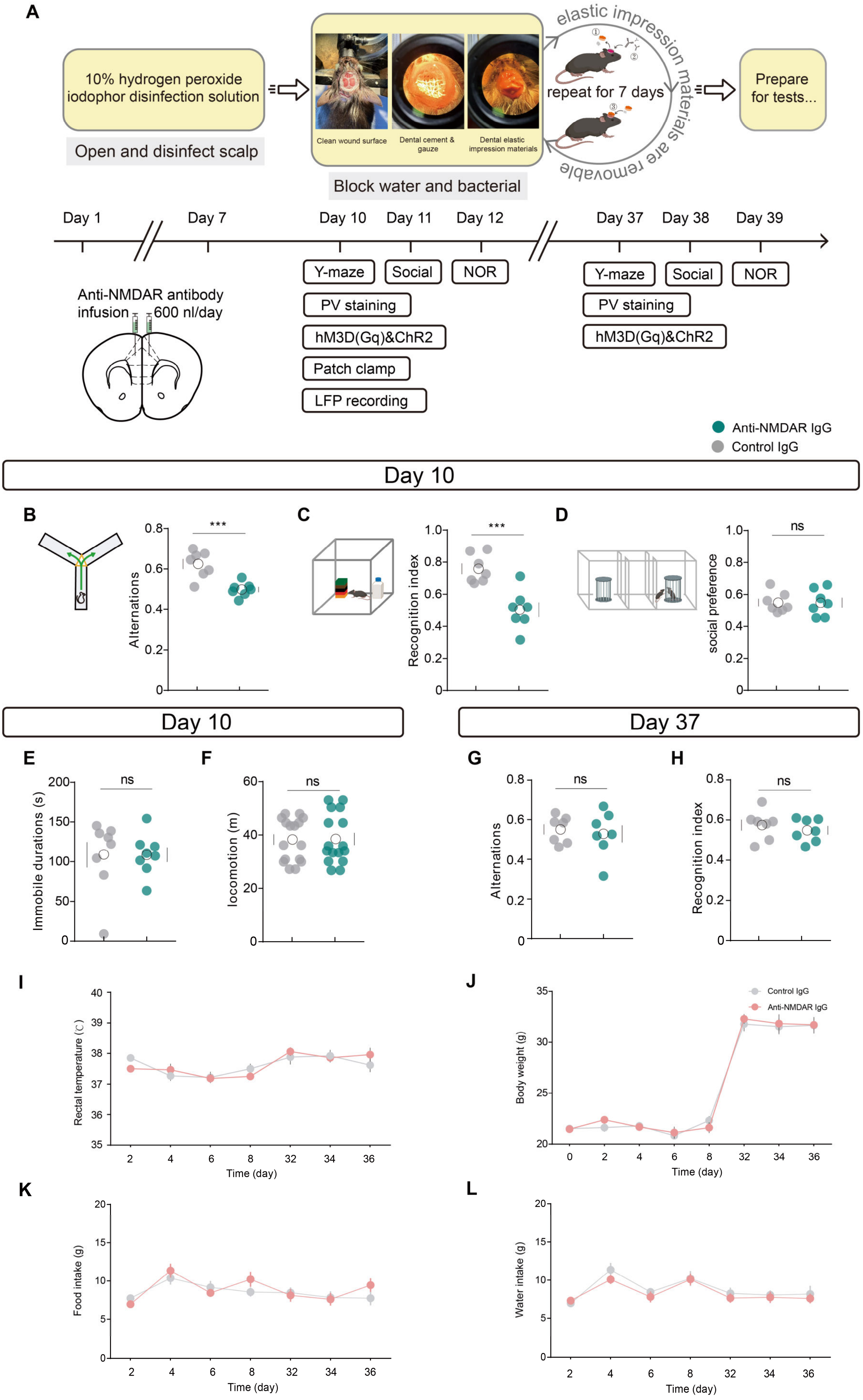
A
10% hydrogen peroxide iodophor disinfection solution
Open and disinfect scalp
Clean wound surface
Dental cement & gauze
Dental elastic impression materials
Block water and bacterial
elastic impression materials are removable
repeat for 7 days
Prepare for tests...
Day 1
Day 7
Day 10
Day 11
Day 12
Day 37
Day 38
Day 39
Y-maze
Social
NOR
PV staining
hM3D(Gq)&ChR2
Patch clamp
LFP recording
Anti-NMDAR antibody infusion 600 nl/day
Anti-NMDAR IgG
Control IgG
Day 10
B
C
D
Alternations
Recognition index
social preference
***
ns
Day 10
Day 37
E
F
G
H
Immobile durations (s)
locomotion (m)
I
J
K
L
Rectal temperature (°C)
Body weight (g)
Food intake (g)
Water intake (g)
Time (day)

Figure S2. Injections of control/anti-NMDAR IgG in the medial prefrontal cortex led to cognitive impairment in mice, without affecting their metabolic status. (**A**) Experimental design. (**B-F**) After 7 days of antibody injection, the alternations(**B**), recognition index(**C**), social preference (**D**), immobile durations (**E**), and locomotion (**F**) of mice. (**G-H**) 30 days after antibody injection, the alternations (**G**) and recognition index (**H**) of mice. $n = 8$ for (E), $n = 16$ for (F), $n = 7$ for the remaining. $^{***}P < 0.001$. (**I-L**) During and after antibody injection, the rectal temperature (**I**), body weight (**J**), food intake (**K**) and water intake (**L**) of the mice, $n = 6$ for (I-L).

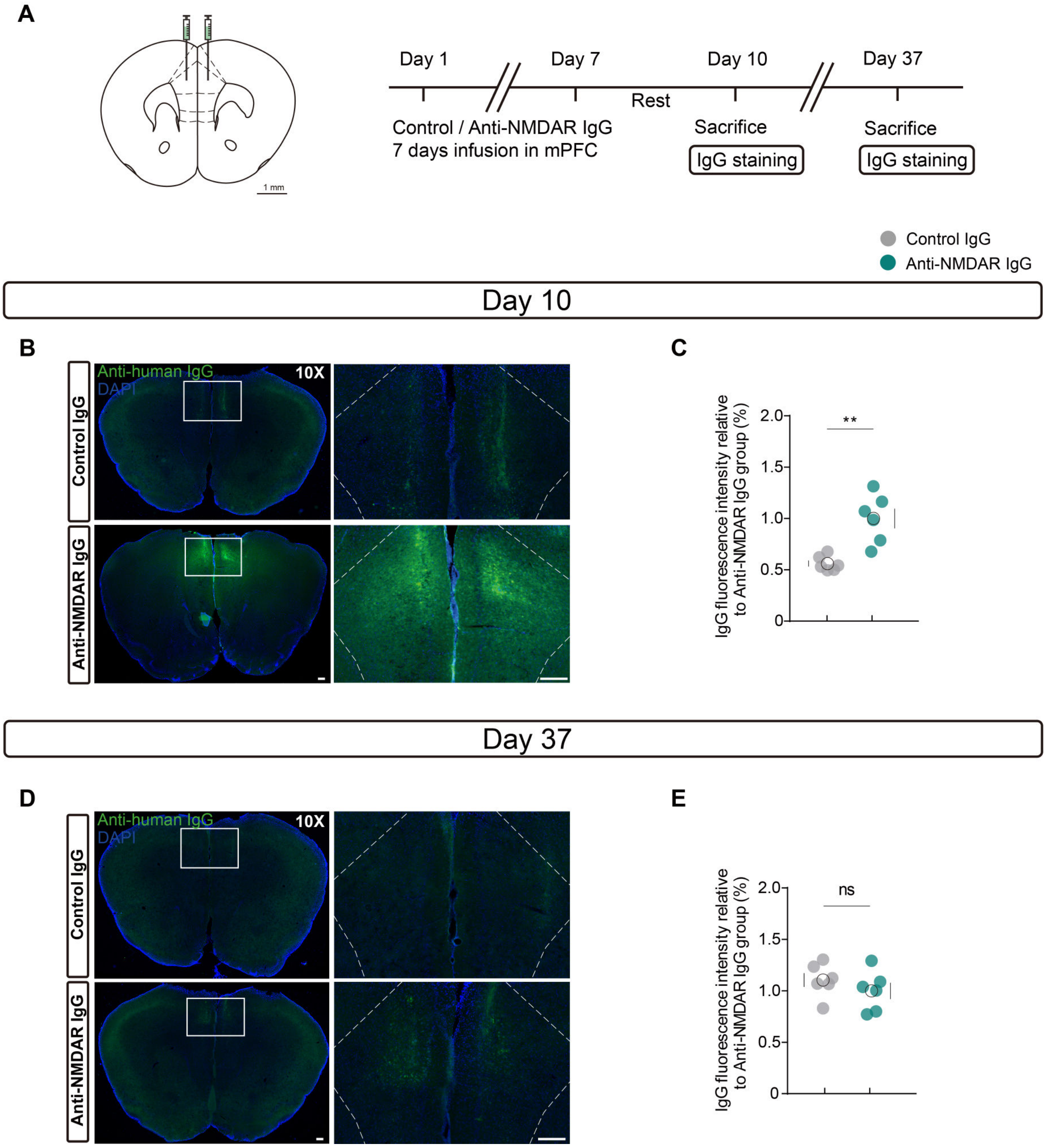

Figure S3. Binding of the anti-NMDAR IgG to the brain region is reversible. (**A**) Experimental design. Scale bar = 1 mm. (**B-E**) IgG fluorescence intensity of anti-NMDAR IgG injection group and control group at 7 (**B-C**) and 30 (**D-E**) days. Scale bar = 200 μm, *n* = 6. $^{**}P$ <0.01.

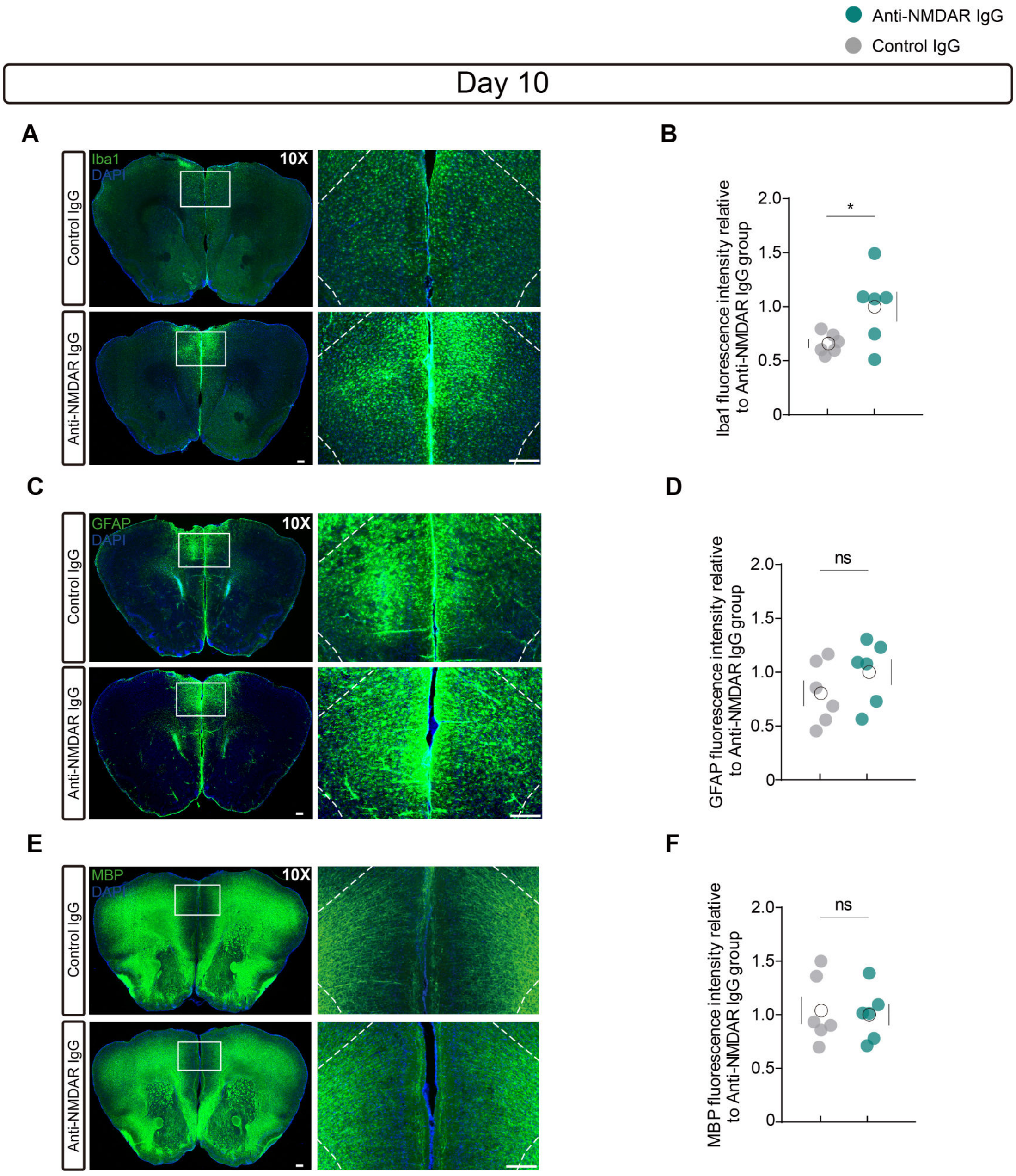

Figure S4. The inflammatory impact induced by anti-NMDAR IgG injection in the mPFC. (**A-C**) Changes of microglia (**A**), astrocytes (**B**) and oligodendrocytes(**C**) induced by injection of anti-NMDAR IgG or control IgG in the mPFC. Scale bar = 200 μm. n = 6. $^{*}P$<0.05.

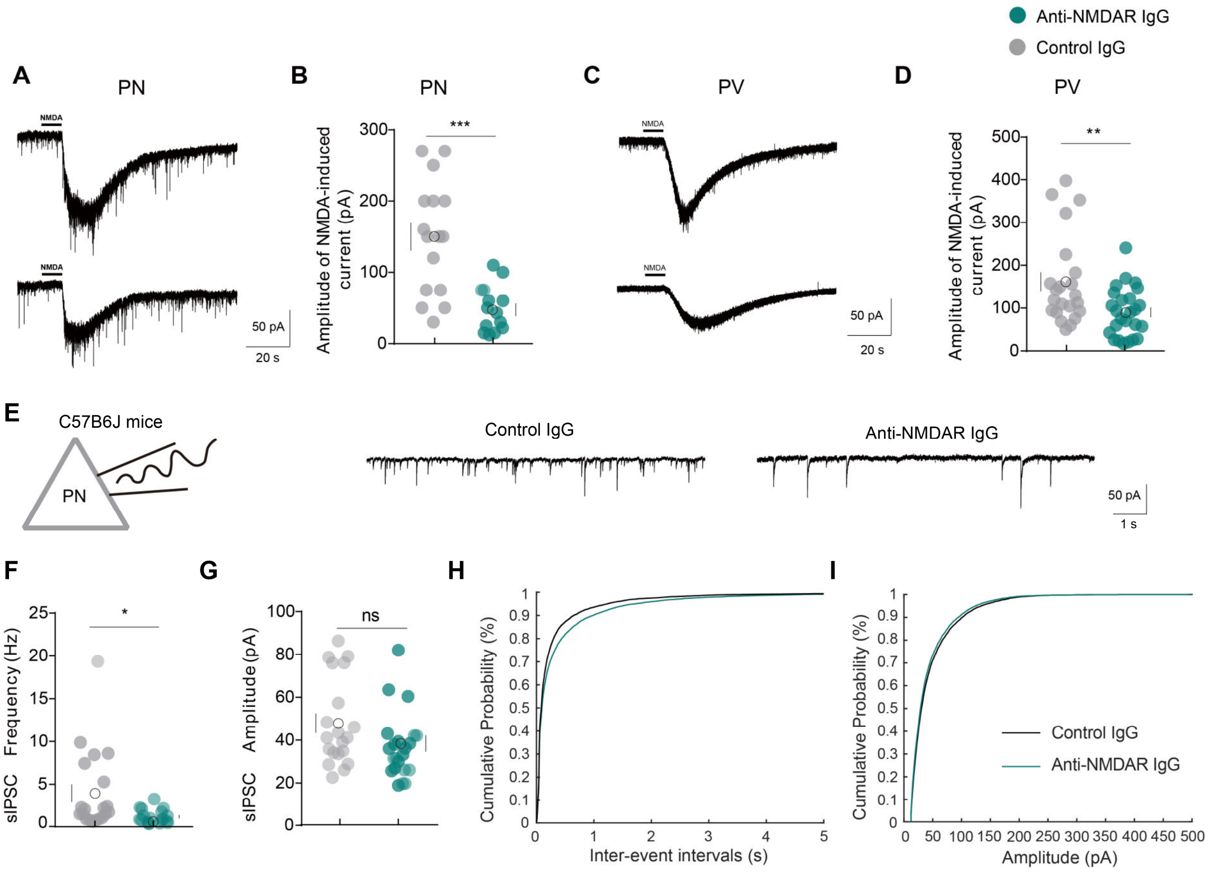

Figure S5. Anti-NMDAR IgG attenuates inhibitory inputs to mPFC pyramidal neurons and equivalently reduces NMDA currents in both pyramidal and PV neurons.

(**A-D**) Sample response of pyramidal (**A**) and PV (**C**) neurons to rapid application of NMDA. (**B**) The amplitude of NMDA currents was significantly reduced in pyramidal neurons following antibody injection, n = 13-16 neurons from 6 mice, $^{***}P < 0.001$. (**D**) The amplitude of NMDA currents was significantly reduced in PV neurons following antibody injection, n = 22-25 neurons from 6 mice, $^{**}P < 0.01$. (**E**) Representative traces of IPSCs of PV neurons. (**F**) Average frequency of sIPSCs in mice injected with control or anti-NMDAR IgG, n = 18-20 neurons from 6 mice, $^{*}P < 0.05$. (**G**) Average amplitude of sIPSCs in mice injected with anti-NMDAR or control IgG, n = 18-20 neurons from 6 mice. (**H**) Cumulative probability plots of the inter-event interval of IPSCs, analyzed using Kruskal-Wallis one-way ANOVA. (**I**) Cumulative probability plots of the amplitude of IPSCs, analyzed using Kruskal-Wallis one-way ANOVA.

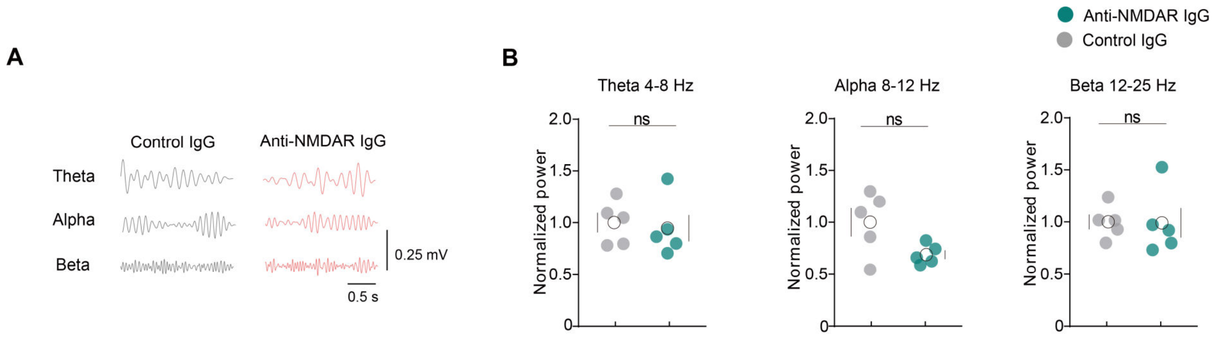

Figure S6. Injection of anti-NMDAR IgG did not result in statistically significant differences in theta, alpha, or beta frequency bands. (**A**) Example LFP data across different frequency bands in mice treated with anti-NMDAR IgG and control IgG. (**B**) Statistical analysis of normalized LFP power in the theta, alpha, and beta bands. $n = 5$.

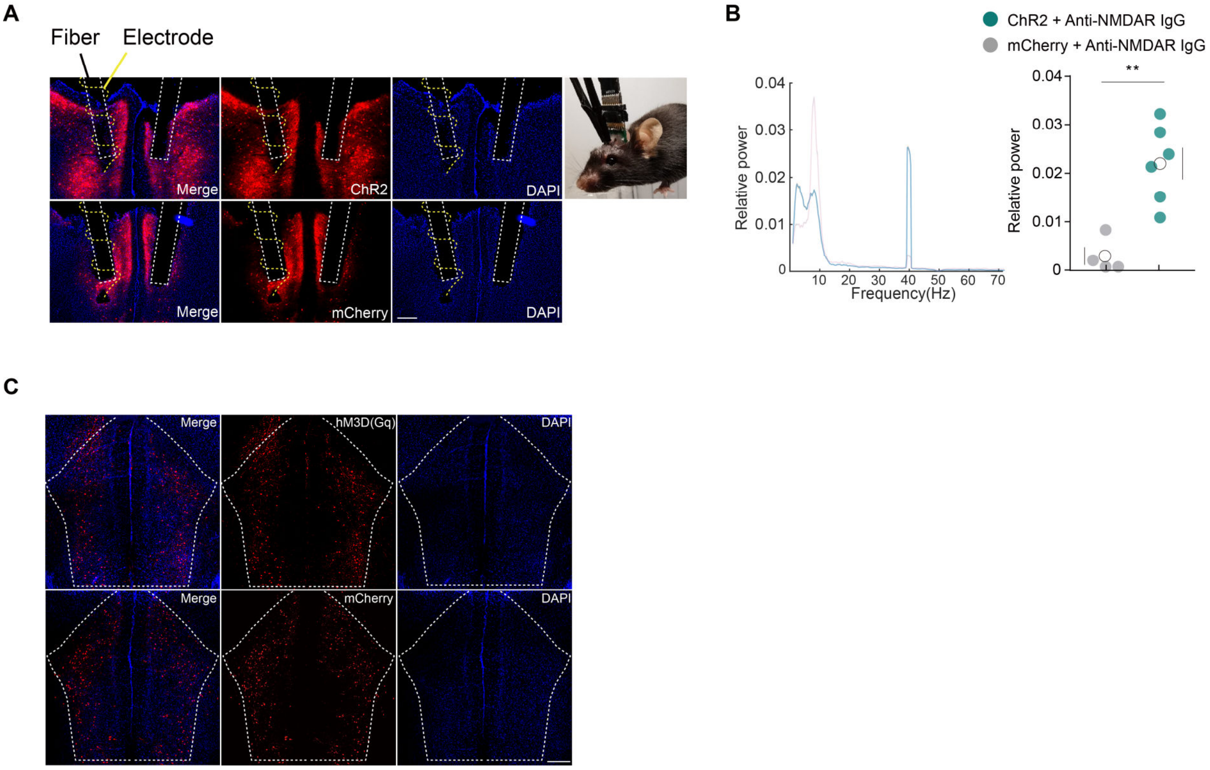

Figure S7. Validation of the expression of optogenetic and pharmacogenetic viruses. (**A**) Schematic diagram of optogenetic virus expression in the mPFC. (**B**) Enhanced gamma oscillations generated by 40 Hz light stimulation, n = 4-6, $^{**}P$ <0.01. (**C**) Schematic diagram of chemogenetic virus expression in the mPFC. Scale bar = 200 μm.

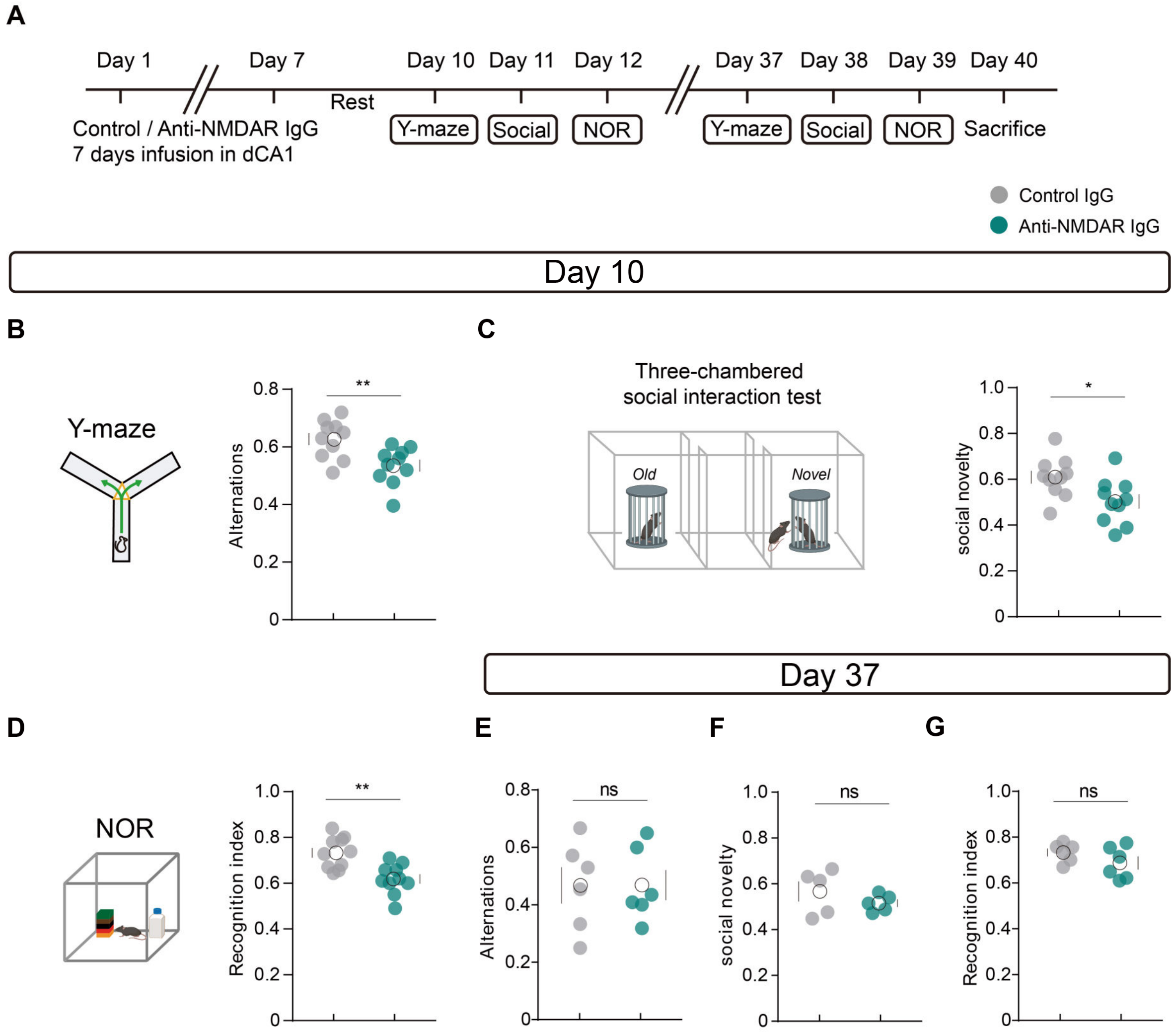

Figure S8. Behavioral test results after injection of anti-NMDAR IgG in the dCA1 region. (A) Schematic of experimental design. **(B-D)** Mice injected with anti-NMDAR IgG exhibited cognitive impairments in the Y-maze test (**B**), social three-chamber test (**C**), and NOR test (**D**). n = 10. $^{*}P<0.05$, $^{**}P<0.01$. **(E-G)** Thirty days later, the cognitive impairments in mice injected with anti-NMDAR IgG had recovered, in the Y-maze test (**E**), social three-chamber test (**F**), and NOR test (**G**) n = 5-6.

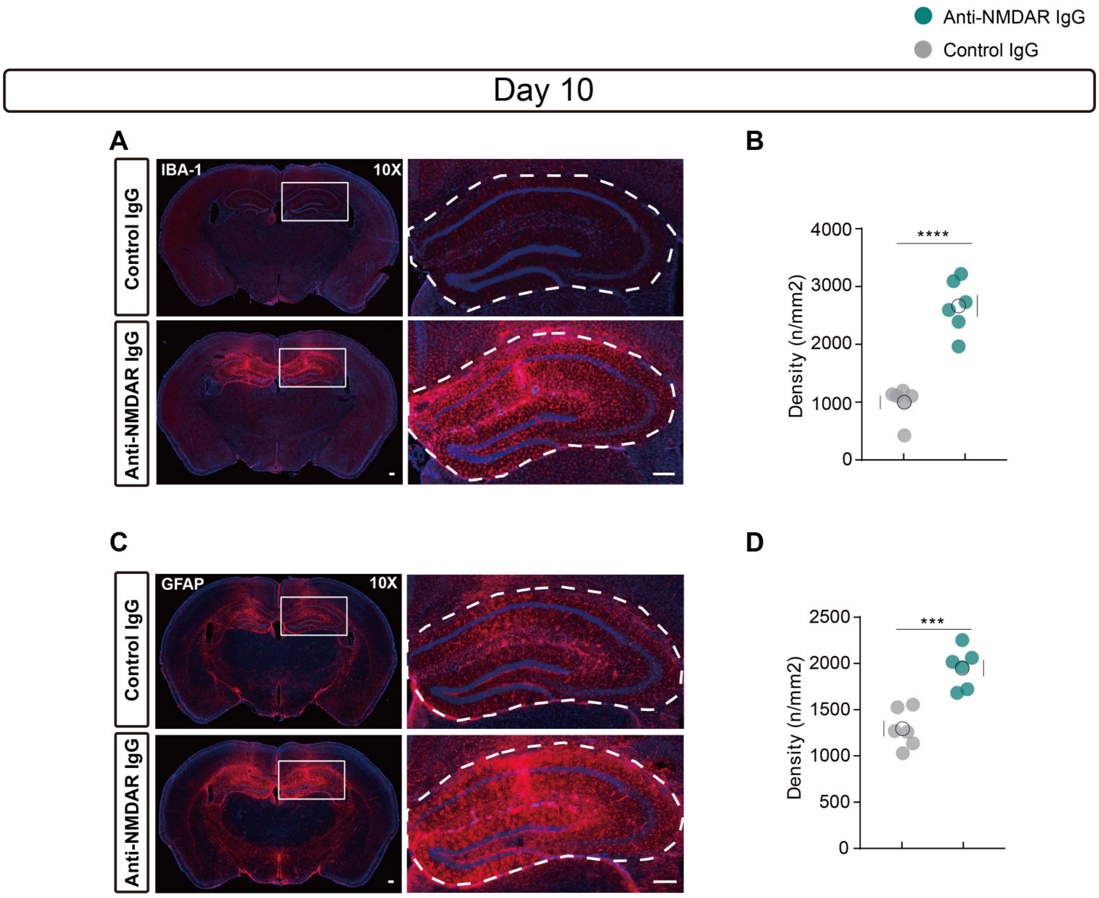

Figure S9. Injection of anti-NMDAR IgG for seven days in the CA1 region led to neuroinflammation. (**A-D**) Immunofluorescence staining showed significant differences in IBA-1 (**A-B**) and GFAP (**C-D**) after injection of anti-NMDAR IgG. $n = 6$, $^{***}P<0.001$, $^{****}P<0.0001$. Scale bar = 200 μm.

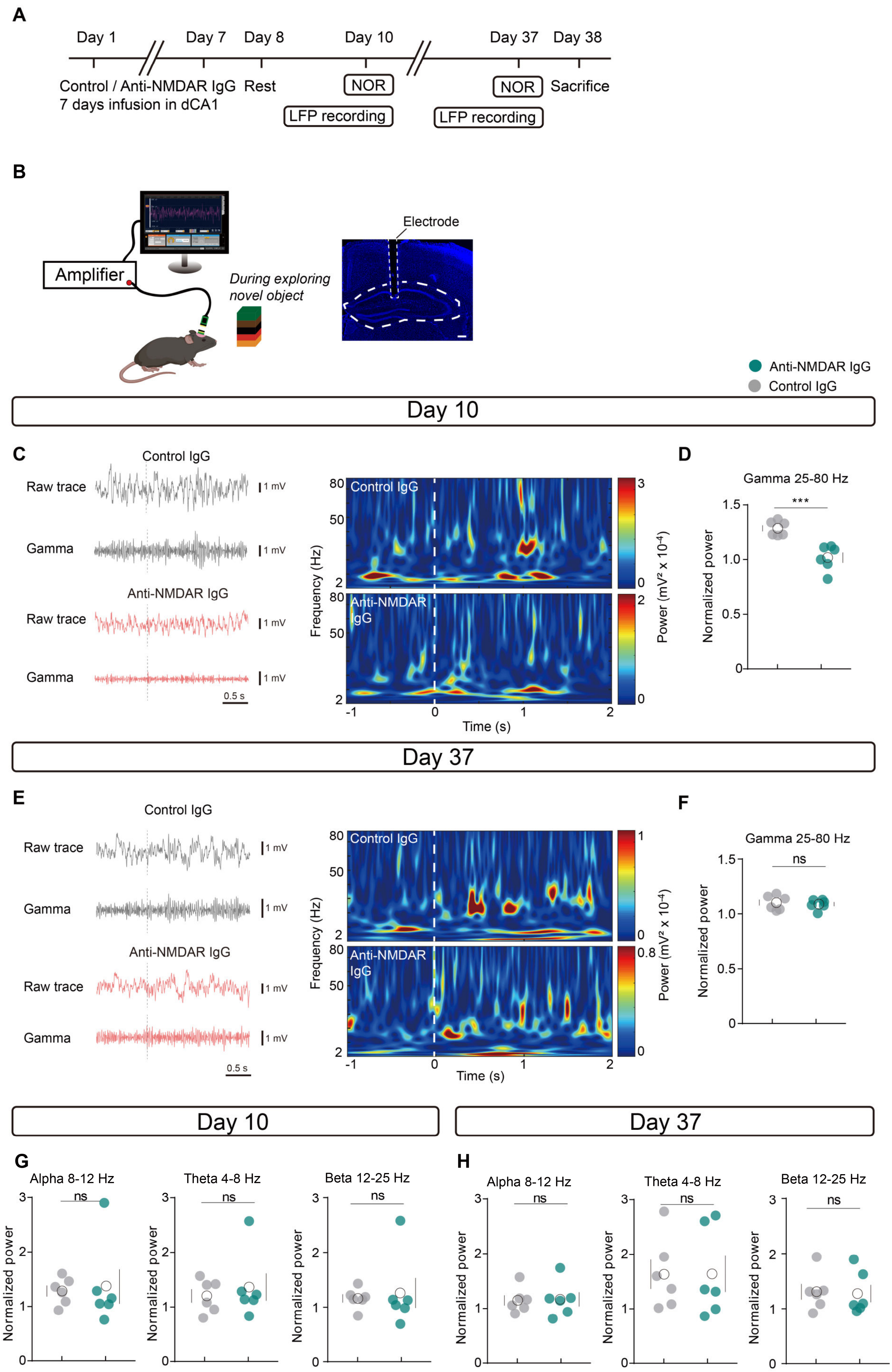
A
Day 1
Day 7
Day 8
Day 10
Day 37
Day 38
Control / Anti-NMDAR IgG
7 days infusion in dCA1
Rest
NOR
LFP recording
NOR
Sacrifice
LFP recording
B
Amplifier
During exploring novel object
Electrode
Anti-NMDAR IgG
Control IgG
Day 10
C
Control IgG
Raw trace
Gamma
Anti-NMDAR IgG
Raw trace
Gamma
1 mV
0.5 s
Frequency (Hz)
Time (s)
Power (mV² x 10⁻⁴)
D
Gamma 25-80 Hz
***
Normalized power
Day 37
E
Control IgG
Raw trace
Gamma
Anti-NMDAR IgG
Raw trace
Gamma
F
Gamma 25-80 Hz
ns
Day 10
Day 37
G
Alpha 8-12 Hz
Theta 4-8 Hz
Beta 12-25 Hz
ns
H
Alpha 8-12 Hz
Theta 4-8 Hz
Beta 12-25 Hz
ns

Figure S10. The injection of anti-NMDAR IgG in the hippocampus dCA1 caused cognitive impairment and decreased gamma oscillations. (**A**) Experimental design of control/anti-NMDAR IgG injection in the hippocampus dCA1. (**B**) Experimental process and optical fiber position. Scale bar = 200 μm. (**C-D**) After seven days injection of anti-NMDAR IgG, gamma (25-80 Hz) oscillations are abnormal in the group of mice that received patients' antibodies. (**E-F**) Thirty days after injection of anti-NMDAR IgG, the abnormal gamma oscillations spontaneously recover. (**G-H**) On day 7 and 30 after antibody injection, there are no significant differences in Alpha (8-12 Hz), Theta (4-8 Hz), and Beta (12-25 Hz) oscillations. $n = 6$. $^{***}P < 0.001$.

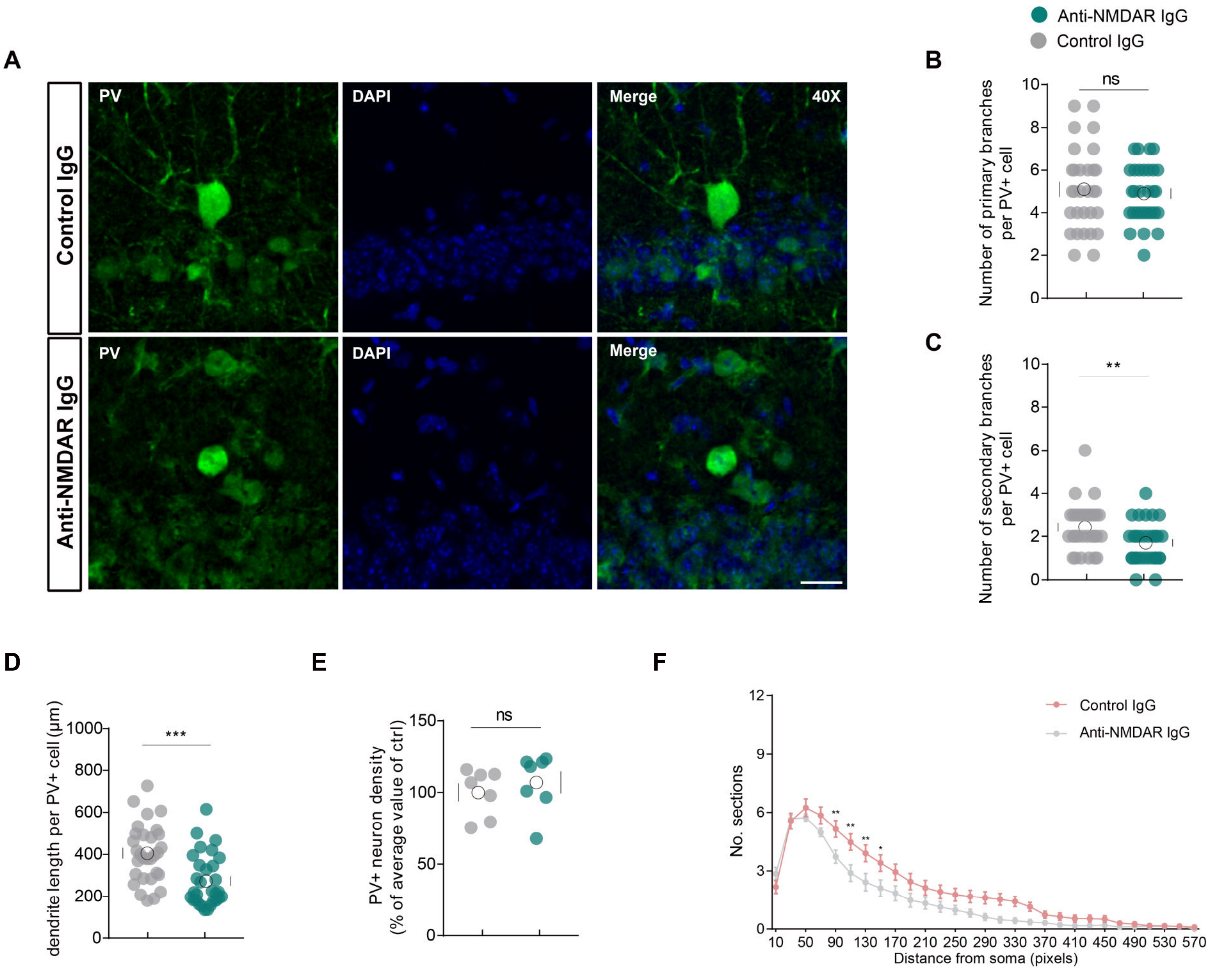

Figure S11. The injection of anti-NMDAR IgG in the hippocampus dCA1 caused changes in PV neurons. (**A-F**) 7 days after antibody injection, the morphology, scale bar = 20 μm. (**A**), number of primary branches (**B**), dendrite length (**C**), number of secondary branches (**D**), PV+ neuron density (**E**) and Sholl analysis (**F**) of PV neurons. 1 μm = 3.48 pixels, *n* = 7 mice for PV+ neuron density (E), *n* = 30-33 neurons from 7 mice for the remaining. $^{*}P<0.05$, $^{**}P<0.01$, $^{***}P<0.001$.
